# Design, Synthesis, and Biological Evaluation of Highly Functionalized Tetrahydro-β-carboline-imidazolium Hybrids Targeting Cholinesterases

**DOI:** 10.3390/molecules31101563

**Published:** 2026-05-08

**Authors:** Agnieszka Hryniewicka, Damian Pawelski, Marta Eliza Plonska-Brzezinska

**Affiliations:** Department of Organic Chemistry, Faculty of Medicine, Division of Dentistry and Division of Medical Education in English, Medical University of Bialystok, Mickiewicza 2A, 15-222 Bialystok, Polandmarta.plonska-brzezinska@umb.edu.pl (M.E.P.-B.)

**Keywords:** Alzheimer’s disease, acetylcholinesterase, butyrylcholinesterase, hybrid imidazolium salt, tetrahydro-β-carboline

## Abstract

A novel series of hybrid tetrahydro-β-carboline (THβC)-imidazolium (IM) salts incorporating a fused diketopiperazine scaffold was designed, synthesized, and evaluated as cholinesterase inhibitors for potential application in Alzheimer’s disease. The molecular design integrates a π-conjugated THβC core with a cationic IM moiety to promote dual-site interactions within the acetylcholinesterase (AChE) active-site gorge. All compounds exhibited micromolar inhibitory activity against AChE and butyrylcholinesterase (BChE), with a pronounced preference for AChE. The most active derivative, **12d**, showed an IC_50_ value of 0.72 μM toward AChE, while compound **12c** demonstrated the highest selectivity (SI = 8.4). Structure–activity relationship studies revealed that both stereochemistry and *N*-alkyl chain length are critical determinants of activity, with *S*,*S*-configured derivatives consistently outperforming their *R*,*R*-configured analogs. In silico ADMET analysis indicated favorable physicochemical properties and predicted central nervous system permeability, although potential hepatotoxicity highlights the need for further optimization. Molecular docking studies suggested that the most promising compound adopts a dual-binding mode, interacting with both the peripheral anionic site and catalytic active site of AChE. These results identify THβC-IM hybrids as a structurally novel and promising scaffold for the development of selective cholinesterase inhibitors, providing a basis for further optimization toward multifunctional anti-Alzheimer agents.

## 1. Introduction

Alzheimer’s disease (AD) is a progressive neurodegenerative disorder characterized by cognitive decline, extracellular accumulation of amyloid-β (Aβ) plaques, and intracellular neurofibrillary tangles composed of hyperphosphorylated tau protein [1]. It represents the most common cause of dementia worldwide, posing a significant and growing burden on healthcare systems due to population aging. Despite extensive research efforts, currently approved therapies provide only symptomatic relief and fail to halt or reverse disease progression [2].

In recent years, THβC derivatives have attracted considerable attention owing to their structural diversity and wide range of biological activities [3]. The THβC scaffold is commonly found in both natural and synthetic compounds that exhibit diverse pharmacological effects, including anticancer [4,5,6], antimicrobial [7], antimalarial [8], antiviral [9], and antifungal activities [10,11]. β-Carboline-based natural products are of significant importance in medicinal and synthetic chemistry, while 1,3-disubstituted THβC derivatives represent a particularly valuable group of structurally and biologically relevant analogs [12]. These compounds exhibit a broad spectrum of pharmacological properties, especially in the context of neurological disorders [13], and several derivatives have been identified as effective inhibitors of acetylcholinesterase (AChE) and butyrylcholinesterase (BChE) [14,15]. Notably, numerous THβC derivatives exhibit neuroprotective effects [16], including antioxidant activity [17] and the ability to modulate cholinesterases such as AChE and BuChE [18]. These mechanisms are highly relevant to the pathogenesis of Alzheimer’s disease, in which oxidative stress, cholinergic dysfunction, and neurotransmitter imbalance play crucial roles [19]. Furthermore, selected THβC-based compounds have been shown to inhibit Aβ aggregation [20] and exhibit anti-neuroinflammatory activity [21], further supporting their therapeutic potential. Among THβC derivatives, tadalafil is one of the most extensively studied compounds, clinically used to treat erectile dysfunction [22] and pulmonary arterial hypertension [23]. Recent studies by Li et al. have focused on the design of novel tadalafil analogs as dual-target inhibitors of AChE and phosphodiesterase type 5 (PDE5), highlighting their potential as candidate therapeutics for Alzheimer’s disease [24,25].

Imidazolium salts (IMSs), a class of ionic imidazole-based compounds, have attracted considerable attention due to their diverse biological activities [26]. Among these, their ability to inhibit AChE has been widely documented for both IM [27,28,29] and benzimidazolium [30,31] derivatives. Furthermore, these compounds have been reported to exert neuroprotective effects by modulating the aggregation behavior of β-amyloid (Aβ42), a peptide implicated in the pathogenesis of Alzheimer’s disease [32,33,34]. Available structure–activity relationship data suggest that inhibitory potency toward AChE is influenced by structural features such as the presence of a cationic nitrogen center and the hydrophobic character of substituents. In particular, elongation of the alkyl chain is associated with enhanced activity, which may result from improved interactions within the enzyme environment [35]. The active site of AChE is located deep within a narrow, predominantly hydrophobic gorge and comprises both catalytic and anionic regions. The latter is responsible for binding the quaternary ammonium group of the natural substrate [36]. IMSs, due to their positive charge, may act as substrate analogs and interact with this region through electrostatic and non-covalent interactions. Increased lipophilicity likely facilitates access to the active site and stabilizes binding within the gorge [35]. In addition to their biological efficacy, IMSs have been evaluated for safety. Experimental evidence indicates that selected representatives do not induce detectable DNA damage or membrane disruption in human leukocyte cells under standard assay conditions, suggesting a relatively low genotoxic risk [37].

The present study encompasses the design, synthesis, and biological evaluation of a new class of THβC-IM hybrids as potential therapeutic agents for Alzheimer’s disease. The combination of the THβC scaffold with quaternized IM moieties represents a rational and innovative strategy for developing novel cholinesterase inhibitors with enhanced therapeutic potential [38]. Such molecular hybridization is anticipated to improve key pharmacological features, including blood–brain barrier permeability and overall pharmacokinetic properties, thereby addressing important limitations of existing therapies for neurodegenerative diseases. There is one reported example in the literature describing the synthesis and antitumor activity of THβC-benzimidazolium salts [39]. However, to the best of our knowledge, the compounds presented in this study represent the first class of hybrid systems integrating an IM-based cationic moiety with the THβC core, further extended by a fused diketopiperazine scaffold that enforces a defined spatial organization and conformational rigidity. This unique combination introduces a new structural motif that has not been previously reported in the context of THβC-derived hybrid architectures.

## 2. Results

### 2.1. Design of Hybrid THβC-IMSs

**Figure 1:**
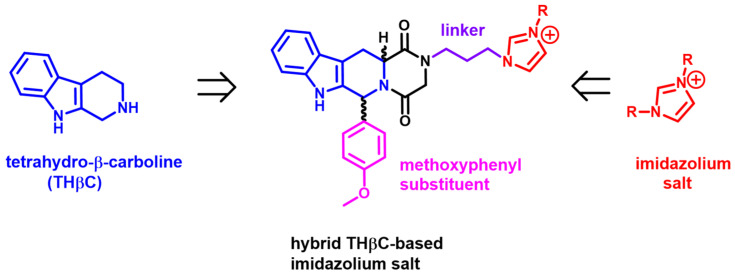
Design strategy of hybrid THβC-based IMSs as AChE/BChE inhibitors.

The design of the investigated compounds was based on a hybrid molecular approach combining structural features of β-carboline derivatives with IMSs, both of which are known to exhibit biological activity relevant to cholinesterase inhibition [40] (Figure 1).

The β-carboline scaffold was selected due to its rigid, planar, and π-conjugated structure, which enables efficient π-π stacking interactions within the aromatic residues lining the active-site gorge of cholinesterases [3]. This feature is particularly important for AChE, whose active site is located at the bottom of a narrow and hydrophobic gorge enriched with aromatic amino acids [36]. The incorporation of the quaternized IM moiety introduces a permanent positive charge, which is expected to enhance binding affinity through electrostatic interactions with the anionic subsite of the enzyme [35]. This positively charged fragment may mimic the quaternary ammonium group of the natural substrate (acetylcholine), facilitating recognition and anchoring within the catalytic gorge [36]. Additionally, the IM unit can contribute to improved aqueous solubility and favorable pharmacokinetic properties [41]. The presence of a flexible linker between the β-carboline core and the IM fragment allows for optimal spatial arrangement of the pharmacophores, enabling simultaneous interactions with multiple regions of the enzyme, including both the catalytic active site (CAS) and the peripheral anionic site (PAS) [42]. Such dual-binding capability is a well-established strategy in the design of effective cholinesterase inhibitors, as it may enhance inhibitory potency and modulate amyloid aggregation processes [43]. Furthermore, variation in the *N*-substituent on the IM ring was introduced to modulate lipophilicity and steric properties, thereby influencing penetration into the enzyme gorge and overall binding strength [44]. This structural tuning enables the exploration of structure–activity relationships and optimization of both potency and selectivity. The selection of a *p*-methoxyphenyl substituent at the C-6 position was based on studies by Li et al., which demonstrated that this functional group enhances inhibitory activity toward AChE [25].

An important structural aspect of the studied hybrids is the stereochemical configuration of the β-carboline core, which differentiates series derived from D-tryptophan (configuration *R*,*R* on two stereogenic centers in the IMS structure) from L-tryptophan derived series (configuration *S*,*S*). This variation was intentionally introduced to evaluate the impact of stereochemistry on cholinesterase inhibition. Overall, the β-carboline-IM hybrid architecture represents a rational design strategy focused on combining complementary interaction modes: π-π stacking, electrostatic attraction, and hydrophobic effects, to achieve effective and selective cholinesterase inhibition.

### 2.2. Chemistry

**Scheme 1:**
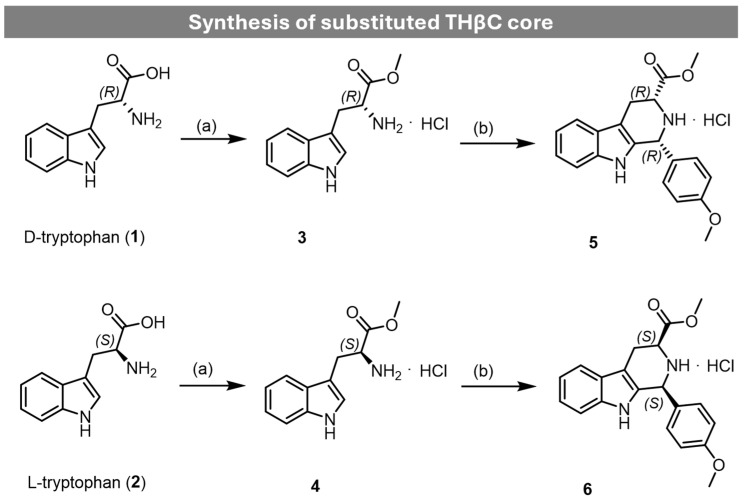
Reaction conditions: (a) SOCl_2_, MeOH, 0–50 °C, 24 h; (b) 4-methoxybenzaldehyde, benzoic acid, MeNO_2_, reflux, 12 h.

Two series of THβC-based IMSs, differing in the configuration of two stereogenic centers, were synthesized starting from D- and L-tryptophan (**1** and **2**, respectively) (Scheme 1). The first stereogenic center originated from the amino acid precursor, while the second was introduced during the subsequent synthetic transformations.

**Scheme 2:**
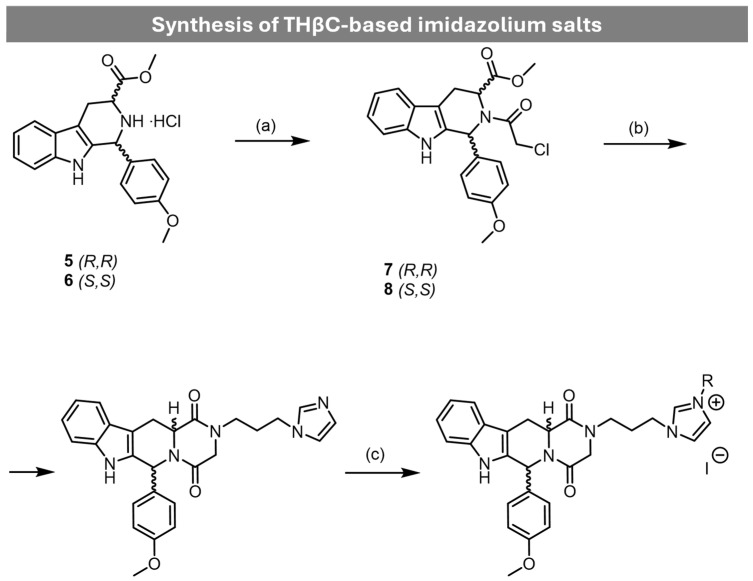
Reaction conditions: (a) chloroacetyl chloride, Et_3_N, DCM, 0 °C, 1 h; (b) 1-(3-aminopropyl)imidazole, DMF:H_2_O 8:2, MW 120 °C, 2 h; (c) RI (neat), MW 110 °C, 5 min.

Following esterification, D- or L-tryptophan was converted into the corresponding methyl esters (**3** and **4**). These intermediates were subjected to a Pictet-Spengler reaction with anisaldehyde in nitromethane, leading to the formation of THβC derivatives (**5** and **6**) through expansion of the indole ring system (Scheme 1) [45]. The addition of a catalytic amount of benzoic acid improved the reaction yield to 93% for **5** and 92% for **6** and enhanced stereoselectivity. As the reaction yields two diastereomers (*cis* and *trans*), their configuration was assigned based on ^1^H NMR chemical shift analysis and comparison with literature data [46]. The *cis* isomer was identified as the major product and preferentially precipitated from the reaction mixture due to its lower solubility in nitromethane. In the next step, acylation of the secondary amine functionality was carried out by reaction of **5** and **6** with chloroacetyl chloride in the presence of triethylamine, affording intermediates **7** and **8** in high yields (90% and 88%, respectively) (Scheme 2). The structures of these compounds were carefully analyzed. It was expected that intermediates **7** and **8** should be *cis* isomers, since they were obtained from the corresponding *cis* isomers **5** and **6**. The general method for assigning the stereochemistry of 1,3-disubstituted-1,2,3,4-tetrahydro-β-carbolines was adopted [47]. According to this approach, the ^13^C NMR signals for C-1 and C-3 in the *trans* diastereomers appear significantly upfield relative to those of the corresponding *cis* isomers. In the literature, the ^13^C NMR spectrum of the *cis* isomer of methyl 2-(2-chloroacetyl)-1-(4-methoxyphenyl)-2,3,4,9-tetrahydro-1*H*-pyrido[3,4-b]indole-3-carboxylate shows signals at 59.8 and 55.6 ppm, whereas for the *trans* isomer these appear at 55.2 and 52.3 ppm, respectively [48]. The NMR spectra of compounds **7** and **8** are very similar, indicating that only one stereoisomer is present in each case. The chemical shifts in C-1 and C-3 (58.8 and 56.6 ppm for **7**, and 58.0 and 56.3 ppm for **8**) confirm that both compounds are *cis* isomers (**7**—C3-*R*, C1-*R* and **8**—C3-*S*, C1-*S*). In the next step, compounds **7** and **8** were reacted with 1-(3-aminopropyl)imidazole, serving as a source of the imidazole moiety with a three-carbon linker, to yield the corresponding 2,3,6,7,12,12a-hexahydropyrazino[1′,2′:1,6]pyrido[3,4-b]indole-1,4-dione derivatives (**9** and **10**). The transformation proceeded via nucleophilic substitution (S_N_2) of the chlorine atom by a primary amine, followed by intramolecular cyclization (6-endo-trig). Optimal results were obtained with a twofold molar excess of the amine, as HCl generated during the reaction reduced nucleophilicity by protonating unreacted amine. Attempts to replace the amine with a mild base (K_2_CO_3_ or triethylamine) resulted in complex reaction mixtures and a significant decrease in isolated yields. Efficient conversion required microwave (MW) irradiation, and the final products were obtained in good yields of approximately 87% for both imidazoles **9** and **10**.

**Figure 2:**
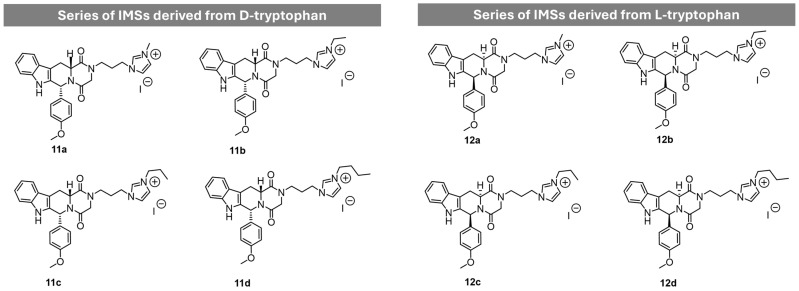
THβC-based IMSs.

The final step in the synthesis of THβC-IMSs involved the alkylation of IM derivatives **9** and **10** with a series of alkyl iodides varying in chain length. Methyl, ethyl, propyl, and butyl iodides were employed to afford the corresponding IMSs bearing C_1_-C_4_ alkyl side chains, respectively. The reactions were performed under MW irradiation using neat alkyl iodides as both reagent and solvent, completing within 5 min. Then, the resulting salts were precipitated by adding n-hexane, and excess alkyl iodide was removed. High-purity products were subsequently obtained by recrystallization from a dichloromethane/diethyl ether mixture, affording the desired salts in excellent yields (Table 1). Two series of IMSs, differing in configuration and alkyl chain length, are presented in Figure 2.

### 2.3. Cholinesterases Inhibitory Activity

**Figure 3:**
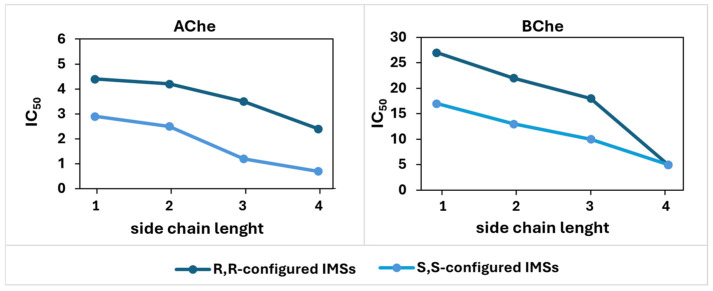
The relationship between side-chain length (expressed as the number of carbon atoms) and IC_50_ values against AChE and BChE for two series of IMSs.

**Figure 4:**
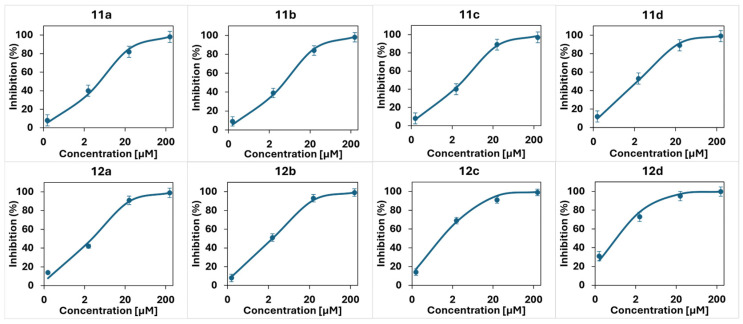
Inhibitory activity of THβC-based IMSs against AChE.

**Figure 5:**
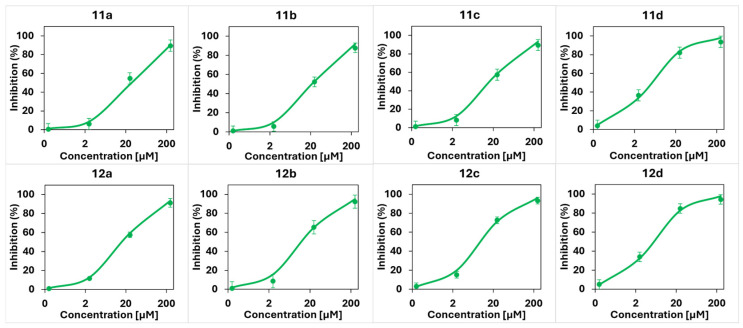
Inhibitory activity of THβC-based IMSs against BChE.

All hybrid THβC-IMS derivatives (8 compounds) were evaluated for the AChE and the BChE inhibitory activities in vitro by the modified Ellman method [49]. The known ChE inhibitor tacrine (**TAC**) was used as a positive reference. The inhibitory activities (Figure 3, Figure 4 and Figure 5) were presented as IC_50_ (μM), and the results were summarized in Table 2. All tested compounds exhibited micromolar inhibitory potency against both enzymes, with a clear preference for AChE over BChE. Within the **11a**–**d** series (*R*,*R* configuration), a gradual increase in activity was observed with elongation of the *N*-alkyl substituent. The IC_50_ values toward AChE decreased from 4.41 μM (**11a**, methyl) to 2.39 μM (**11d**, butyl), indicating enhanced potency with increasing lipophilicity. A similar trend was observed for BChE inhibition, with the selectivity index (SI) decreasing significantly for the butyl derivative, suggesting reduced selectivity at longer chain lengths. A comparable structure–activity relationship was observed for the **12a**–**d** series (*S*,*S* configuration), which generally demonstrated higher potency than their *R*,*R* counterparts.

In particular, compound **12d** (butyl substituted) emerged as the most active inhibitor in this series, with IC_50_ values of 0.72 μM for AChE and 5.23 μM for BChE. Notably, compound **12c** (propyl substituent) showed the highest selectivity toward AChE (SI = 8.4), indicating an optimal balance between potency and selectivity. Overall, the *S*,*S*-configured derivatives (**12a**–**d**) were consistently more active than the corresponding *R*,*R* analogs (**11a**–**d**), highlighting the significant influence of stereochemistry on biological activity (Figure 3). The observed increase in inhibitory potency with longer alkyl chains suggests that enhanced hydrophobic interactions within the enzyme active site play a crucial role in ligand binding.

However, excessive chain elongation may lead to reduced selectivity, as observed for compound **11d**. The *S*,*S*-configured derivatives (series **12a**–**d**) consistently exhibited higher inhibitory potency toward AChE compared to their *R*,*R* counterparts (series **11a**–**d**). This suggests that the spatial arrangement of substituents in the *S*,*S* configuration is more favorable for interactions within the enzyme active-site gorge. In particular, this configuration may allow for more effective alignment of the β-carboline aromatic system with key residues responsible for π-π stacking interactions, while simultaneously positioning the IM moiety for optimal electrostatic interaction with the anionic subsite. In contrast, the *R*,*R*-configured compounds (**11a**–**d**) appear to adopt a less favorable binding orientation, which may limit simultaneous interactions with both the CAS and the PAS. As a result, these derivatives show reduced potency and, in some cases, lower selectivity. The observed stereochemical dependence is consistent with the highly constrained architecture of the cholinesterase active site, where even subtle differences in three-dimensional arrangement can significantly influence binding affinity. Therefore, the superior activity of the *S*,*S* series highlights the importance of stereochemical optimization in the design of dual-binding cholinesterase inhibitors. Ni et al., in their studies on tadalafil derivatives, demonstrated that the stereochemistry at position 6 of the tadalafil parent scaffold is crucial for AChE inhibitory activity, with the 6*R* configuration being the most favorable [25]. In contrast, in our studies, we found that introducing a quaternized IM moiety into a structurally related core appears to alter the structure–activity relationship, with compounds bearing the *S*-configuration exhibiting higher activity. This finding is particularly encouraging, as these *S*,*S*-configured derivatives can be readily obtained from the more accessible and cost-effective L-tryptophan precursor.

In this context, the THβC-IM hybrids presented herein demonstrate a balanced pharmacological profile. Although the inhibitory potency of the most active compound (**12d**, IC_50_ = 0.72 μM) remains lower than that of tacrine, it is comparable to or better than many previously reported THβC derivatives [14] or IMSs [35]. Importantly, the studied compounds exhibit a clear preference for AChE over BChE, with selectivity indices reaching 8.4 (compound **12c**), which is significantly higher than that of tacrine (SI ≈ 1). This improved selectivity may translate into a more favorable safety profile, as selective AChE inhibition is considered beneficial in reducing peripheral side effects associated with non-selective cholinesterase inhibitors. Furthermore, the incorporation of a rigid THβC scaffold combined with a cationic IM moiety provides a unique structural framework that enables dual-site interactions within the enzyme gorge, a feature less commonly observed in simpler β-carboline derivatives [17]. These results indicate that the designed hybrids represent a competitive, structurally novel class of cholinesterase inhibitors, offering advantages in selectivity and rational scaffold design compared to examples in the existing literature.

### 2.4. Drug-Likeness Properties of THβC-IMSs

A computational approach was used to predict ADMET features, and selected results were presented in Table 3 [50].

The ADMET profile of compounds **11a**–**d** and **12a**–**d** was evaluated to assess their drug-likeness and to rationalize the observed cholinesterase inhibitory activity. The ADMET results for compounds with identical alkyl side-chain lengths but differing *R*/*S* configurations showed no significant differences. Therefore, these compounds were grouped in the respective columns of the table. According to Lipinski’s rule [51], all compounds exhibit molecular weights in the range of 484.58–526.66 g/mol, within acceptable limits for CNS-active agents, although values near 500 Da may partially limit permeability. The lipophilicity (logP = 2.58–3.84) increases progressively with elongation of the *N*-alkyl substituent, which correlates well with the observed enhancement of AChE inhibitory activity within both series. Compounds bearing longer alkyl chains (**11d** and **12d**) showed the highest potency, which can be attributed to stronger hydrophobic interactions within the enzyme gorge. At the same time, all derivatives maintain moderate water solubility, indicating a balanced hydrophilic-lipophilic profile favorable for biological activity. Importantly, all compounds exhibit moderate BBB and CNS permeability, consistent with their intended central mechanism of action. The combination of moderate lipophilicity and acceptable polarity suggests that these compounds are capable of reaching the central nervous system, supporting their activity against AChE. Toxicological predictions reveal some limitations. Mutagenicity (Ames toxicity test) is predicted for compounds **11a**, **12a, 11d**, and **12d**. In contrast, intermediate analogs (**11b**, **12b** and **11c**, **12c**) appear non-mutagenic, suggesting that both very short and longer alkyl chains may contribute to increased genotoxic risk. All compounds are predicted to exhibit hepatotoxicity, a potential liability that requires further experimental validation. The predicted oral acute toxicity (LD_50_ ≈ 2.48–2.55 mol/kg) indicates moderate toxicity, while the maximum tolerated dose values are relatively consistent across the series. In the context of the predicted maximum tolerated dose, this suggests a potentially favorable balance between efficacy and safety. Notably, compounds **12c** and **12d** combine favorable ADMET properties with the highest inhibitory potency against AChE, making them the most promising candidates. In particular, **12c** appears to offer the best balance between activity, selectivity, and safety, due to its high potency, lack of predicted mutagenicity, and moderate physicochemical properties.

**Figure 6:**
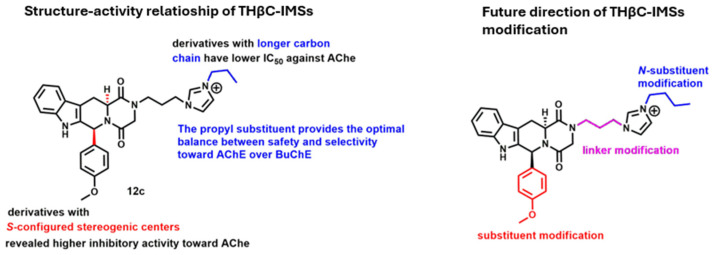
SAR of target compounds and the possibility of modification of THβC-IMS.

Overall, the ADMET analysis supports the structure–activity relationship findings, indicating that increasing lipophilicity enhances biological activity but may simultaneously introduce toxicity-related risks. Therefore, careful optimization of alkyl chain length is required to achieve an optimal balance between efficacy and safety. Although hepatotoxicity was predicted for all compounds, this liability will guide future structural optimization efforts to improve safety profiles while preserving biological activity (Figure 6).

Based on the obtained structure–activity relationship and ADMET data, several directions for further lead optimization can be proposed:

(I) Fine-tuning of the *N*-alkyl substituent on the IM moiety appears critical for balancing potency, selectivity, and toxicity. While elongation of the alkyl chain enhances inhibitory activity, excessive lipophilicity may reduce selectivity and increase toxicity. Therefore, the introduction of branched substituents could improve the pharmacological profile.
(II) Modification of the linker between the THβC core and the IM fragment may allow better spatial positioning within the enzyme gorge, potentially enhancing simultaneous interactions with both the CAS and the PAS. In particular, altering linker length, flexibility, or polarity could further optimize binding efficiency.
(III) Structural variation in the aromatic substituent at the C-6 position represents another promising strategy. Introduction of electron-donating or electron-withdrawing groups may modulate π-π interactions and influence binding affinity.

Finally, given the predicted toxicity liabilities, future efforts should focus on reducing hepatotoxicity through structural simplification, modulation of lipophilicity, or replacement of the IM moiety with alternative cationic or bioisosteric fragments. However, THβC-IMS hybrids provide a clear framework for rational lead optimization toward multifunctional anti-Alzheimer agents.

### 2.5. Molecular Docking Studies

To gain insight into the binding mode of the most promising compound **12c**, molecular docking studies were performed against AChE using SwissDock (Attracting Cavities 2.0) [52,53]. Additionally, **11c** was also analyzed using molecular docking. The best predicted binding pose for **12c** (Cluster 5) exhibited a calculated binding free energy of −7.68 kcal/mol, suggesting a favorable interaction within the enzyme active site. Other clusters showed comparable binding energies (−6.19 to −7.68 kcal/mol), indicating the presence of multiple closely related binding conformations within the AChE gorge. In comparison, **11c** exhibited a calculated binding free energies ranging from −6.05 to −6.79 kcal/mol, indicating weaker interactions within the enzyme active site than **12c**.

**Figure 7:**
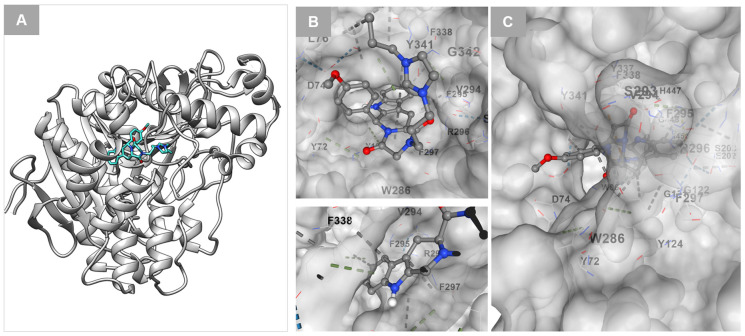
(**A**) Binding mode of **12c** within the AChE gorge; (**B**) Visualization of **12c** interactions with key residue; (**C**) Visualization of **11c** contacts with key residue (lack of interactions with residues at the gorge entrance).

The predicted binding mode was further analyzed using CB-Dock2, which identified a binding cavity corresponding to the canonical active-site gorge of AChE. Contact residue analysis revealed that compound **12c** interacts with amino acid residues distributed along the entire gorge, including the PAS, mid-gorge region, and CAS. Specifically, residues such as Trp286, Tyr124, Tyr72, and Asp74 are associated with the PAS region, while Phe295, Phe297, Val294, and Arg296 line the hydrophobic channel. Importantly, interactions with Phe338, Tyr341, and Gly342 indicate that the ligand extends into the CAS region. As shown in Figure 7A,B, compound **12c** is fully accommodated within the deep catalytic gorge of AChE, adopting an elongated conformation that enables simultaneous interactions with residues from both the PAS and CAS regions. The aromatic moieties of the ligand are oriented toward key residues such as Trp286 and Phe338, facilitating π-π stacking interactions, while the positively charged heterocyclic fragment is positioned in proximity to Tyr341, suggesting the formation of stabilizing π-cation interactions. In addition, multiple hydrophobic contacts with residues lining the gorge contribute to the overall stabilization of the ligand within the binding pocket. Analysis of isomer **11c** revealed that the ligand is primarily stabilized within the CAS, where it forms aromatic π-π interactions with TRP286, PHE295, and tyrosine residues, along with hydrogen bonds. Notably, no interaction with TYR72 at the entrance of the gorge was observed. The *R* configuration of the stereogenic center bearing the methoxyphenyl substituent appears to orient this moiety toward the gorge entrance, where it becomes sterically hindered (Figure 7C). As a result, the ligand does not engage the PAS and remains confined to the inner region of the enzyme.

The observed binding pattern of **12c** is consistent with a dual-site binding mode, characteristic of highly effective AChE inhibitors that span the entire catalytic gorge. Such a binding mode is known to enhance inhibitory potency by simultaneously blocking substrate access at the PAS and interfering with catalytic activity at the CAS. The structural features of compound **12c**, including the presence of aromatic fragments and a positively charged heterocyclic core, appear to support this binding profile. The molecular docking results are in agreement with the experimental enzyme inhibition data, confirming the higher activity of compound **12c** compared to **11c**.

Nevertheless, it should be emphasized that docking results provide a static representation of ligand-protein interactions and rely on simplified scoring functions. Therefore, the proposed binding mode should be considered as a qualitative model. Further validation using molecular dynamics simulations and experimental studies would be necessary to confirm the stability and functional relevance of the predicted interactions.

## 3. Materials and Methods

### 3.1. General Remarks

^1^H and ^13^C NMR spectra were recorded on an Agilent VNMRS system at 500 MHz for ^1^H NMR and 126 MHz for ^13^C NMR. High-resolution mass spectra were acquired using a MALDISynapt G2-S HDMS (Waters Corporation, Milford, MA, USA) coupled to a Waters TQD mass spectrometer (electrospray ionization mode ESI-tandem quadrupole). Fourier transform infrared (FTIR) spectroscopy was performed using a Thermo Scientific Nicolet iN10 MX microscope (Thermo Fisher Scientific, Madison, WI, USA). MW accelerated reactions were performed using a Monowave 450 MW reactor (Anton Paar, Graz, Austria). Methanol was dried over Mg, and DCM was dried over CaH_2_. Ethyl acetate (99,5%), hexane (95%), methanol (99.8%), DCM (99%), DMF (98%) were purchased from Avantor Performance Material, Poland S.A. D-tryptophan (98%), L-tryptophan (98%), *p*-methoxybenzaldehyde (98%), benzoic acid (99,5%), nitromethane (98%), chloroacetyl chloride (98%), triethylamine (99,5%), thionyl chloride (99%), 1-(3-aminopropyl)imidazole (97%), iodomethane (99%), iodoethane (99%), iodopropane (98%), iodobutane (99%) were purchased from Sigma Aldrich and used as received. Copies of NMR spectra of new compounds are included in the Supplementary Materials.

### 3.2. Synthesis Procedures

#### 3.2.1. D-Tryptophan Methyl Ester Hydrochloride (**3**)

Into a 250 mL round-bottomed flask, D-tryptophan (**1**, 10 g, 49 mmol) and anhydrous methanol (200 mL) were added. The suspension was cooled to 0 °C, after which thionyl chloride (10.7 mL, 147 mmol) was added dropwise. The mixture was then heated at 50 °C for 24 h. After this time, the solvent was removed under reduced pressure, ethyl acetate was added to the dry residue, and the resulting suspension was sonicated for 10 min. The precipitate was then filtered off under reduced pressure using a Büchner funnel. The crude compound was finally purified by recrystallization from hot isopropanol, affording D-tryptophan methyl ester hydrochloride as a white solid (11.86 g, 95%). All physical and spectroscopic data are in full agreement with the literature report [54].

#### 3.2.2. L-Tryptophan Methyl Ester Hydrochloride (**4**)

The procedure for the synthesis of ester **3** was followed using L-tryptophan (**2**, 10 g, 49 mmol), anhydrous methanol (200 mL), and thionyl chloride (10.7 mL, 147 mmol) to obtain the product as a white solid (12.23 g, 98%). All physical and spectroscopic data are in full agreement with the literature report [54].

#### 3.2.3. Methyl (1*R*,3*R*)-1-(4-Methoxyphenyl)-2,3,4,9-tetrahydro-1H-pyrido[3,4-b]indole-3-carboxylate Hydrochloride (**5**)

Into a 250 mL round-bottomed flask, D-tryptophan methyl ester hydrochloride (10 g, 39.3 mmol), *p*-methoxybenzaldehyde (6.41 g, 47.1 mmol), benzoic acid (0.1 g, 2 mol%), and nitromethane (200 mL) were added. The reaction mixture was refluxed for 12 h. After this time, the solution was concentrated to half of its original volume under reduced pressure, and the reaction mixture was cooled in an ice bath. The precipitated solid was collected under reduced pressure using a Büchner funnel, and the filtrate was washed with ethyl acetate. The product was obtained as an off-white solid (13.64 g, 93%). All physical and spectroscopic data are in full agreement with the literature report [46].

#### 3.2.4. Methyl (1*S*,3*S*)-1-(4-Methoxyphenyl)-2,3,4,9-tetrahydro-1H-pyrido[3,4-b]indole-3-carboxylate Hydrochloride (**6**)

The procedure for the synthesis of carboxylate **5** was followed with L-tryptophan methyl ester hydrochloride (10 g, 39.3 mmol), *p*-methoxybenzaldehyde (6.41 g, 47.1 mmol), benzoic acid (0.1 g, 2 mol%), and nitromethane (200 mL) to obtain the product as a white solid (13.49 g, 92%). All physical and spectroscopic data are in full agreement with the literature report [46].

#### 3.2.5. Methyl (1*R*,3*R*)-2-(2-Chloroacetyl)-1-(4-methoxyphenyl)-2,3,4,9-tetrahydro-1H-pyrido[3,4-b]-indole-3-carboxylate (**7**)

Into a 250 mL round-bottomed flask, carboxylate **5** (3 g, 8.05 mmol) and anhydrous DCM (100 mL) were added, followed by triethylamine (3.4 mL, 24.1 mmol). The suspension was stirred magnetically for 15 min at 0 °C until it became completely clear. Then, chloroacetyl chloride (0.96 mL, 12.1 mmol) was slowly added, and the reaction mixture was stirred for 1.5 h. The progress of the reaction was monitored by TLC using ethyl acetate as the eluent. After confirming complete conversion of the starting material, the reaction mixture was transferred to a separatory funnel, distilled water was added, and the mixture was acidified to pH ~4 using 10% citric acid. The contents of the funnel were shaken, the organic layer was collected, and the aqueous layer was additionally extracted with DCM. The combined organic layers were washed with saturated NaCl solution (100 mL), dried over anhydrous MgSO_4_, and concentrated under reduced pressure. The dry residue was purified by silica gel column chromatography using a mixture of DCM/ethyl acetate/hexane (2:4:4, *v*/*v*/*v*). The product was obtained as an off-white solid (3.02 g, 90%). ^1^H NMR (500 MHz, acetone-*d*_6_) δ 10.15 (s, 1H), 7.55 (d, *J* = 7.8 Hz, 1H), 7.42 (m, 2H), 7.33 (m, 1H), 7.06 (m, 1H), 7.00 (m, 1H), 6.87 (m, 2H), 6.20 (s, 1H), 5.06 (t, *J* = 4.8 Hz, 1H), 4.55 (d, *J* = 13.7 Hz, 1H), 4.23 (d, *J* = 13.6 Hz, 1H), 3.74 (s, 3H), 3.58 (s, 3H), 3.50 (m, 1H), 3.31 (m, 1H) ppm; ^13^C NMR (125 MHz, acetone-*d*_6_) δ: 171.8, 168.2, 158.9, 136.5, 128.4, 127.4 (2C), 126.2, 121.2, 118.9, 117.8, 114.6 (2C), 113.6, 110.9, 104.9, 58.8, 56.6, 55.3, 55.0, 43.3, 24.0 ppm; ESI-HRMS m/z: calcd for [M+H]^+^ C_22_H_22_ClN_2_O_4_: 413.1268, found 413.1274.

#### 3.2.6. Methyl (1*S*,3*S*)-2-(2-Chloroacetyl)-1-(4-methoxyphenyl)-2,3,4,9-tetrahydro-1H-pyrido[3,4-b]-indole-3-carboxylate (**8**)

The procedure for the synthesis of carboxylate **7** was followed with carboxylate **6** (3 g, 8.05 mmol) and anhydrous DCM (100 mL), anhydrous triethylamine (3.4 mL, 24.1 mmol), and 2-chloroacetyl chloride (0.96 mL, 12.1 mmol) to obtain the product as an off-white solid (2.95 g, 88%). ^1^H NMR (500 MHz, acetone-*d*_6_) δ 10.45 (s, 1H), 7.55 (d, *J* = 7.8 Hz, 1H), 7.42 (m, 2H), 7.33 (m, 1H), 7.06 (m, 2H), 6.87 (m, 2H), 6.20 (s, 1H), 5.06 (t, *J* = 4.8 Hz, 1H), 4.55 (d, *J* = 13.6 Hz, 1H), 4.23 (d, *J* = 13.6 Hz, 1H), 3.74 (s, 3H), 3.58 (s, 3H), 3.48 (m, 1H), 3.34 (m, 1H) ppm; ^13^C NMR (125 MHz, acetone-*d*_6_) δ: 171.9, 168.1, 158.9, 136.7, 128.2, 127.4 (2C), 126.2, 121.2, 118.9, 117.8, 114.6 (2C), 113.6, 111.0, 105.1, 58.8, 56.3, 55.3, 55.0, 43.4, 24.0 ppm; ESI-HRMS m/z: calcd for [M+H]^+^ C_22_H_22_ClN_2_O_4_: 413.1268, found 413.1274.

#### 3.2.7. (6*R*,12a*R*)-2-(2-(1*H*-Imidazoil)propyl)-6-(4-methoxyphenyl)-2,3,6,7,12,12a-hexaahydropyrazino[1′,2′:1,6]pyrido[3,4-b]indole-1,4-dione (**9**)

Into a 30 mL MW vial was added carboxylate **7** (0.50 g, 1.2 mmol), 1-(3-aminopropyl)imidazole (0.3 g, 2.4 mmol), and 15 mL of a DMF/H_2_O mixture (8/2, *v*/*v*). The reaction mixture was heated under MW irradiation at 120 °C for 2 h. Then, the solvents were removed under reduced pressure. The dry residue was purified by flash column chromatography with a MeOH/DCM mixture (2.5/97.5, *v*/*v*). The product was obtained as an off-white solid (0.48 g, 85%). ^1^H NMR (500 MHz, CDCl_3_) δ 8.72 (s, 1H), 7.54 (s, 1H), 7.52 (d, *J* = 7.8 Hz, 1H), 7.31 (d, *J* = 8.0 Hz, 1H), 7.17 (m, 4H), 6.99 (s, 1H), 6.97 (s, 1H), 6.96 (s, 1H), 6.79 (d, *J* = 8.7 Hz, 2H), 4.32 (dd, *J* = 11.9, 4.1 Hz, 1H), 4.07 (d, *J* = 17,5 Hz, 1H), 4.00 (t, *J* = 7 Hz, 2H), 3.91 (d, *J* = 17.5 Hz, 1H) 3.74 (s, 3H), 3.53 (dd, *J* = 15.4, 4.2 Hz, 1H), 3.38 (m, 2H), 2.96 (t, J = 7.1 Hz, 1H), 2.05 (m, 2H) ppm; ^13^C NMR (126 MHz, CDCl_3_) δ 165.8, 161.4, 159.9, 137.0, 136.4, 130.4, 130.1, 129.2, 126.2, 122.6, 120.0, 118.8, 118.3, 114.2, 111.3, 108.5, 55.4, 52.5, 51.7, 49.6, 44.6, 43.6, 28.1, 27.7 ppm; IR (ATR) ν = 2938, 2841, 1645, 1608, 1509, 1451, 1326, 1246, 1174, 1153, 1033, 841, 741 cm^−1^; ESI-HRMS m/z: calcd for [M+H]^+^ C_27_H_28_N_5_O_3_: 470.2192, found 470.2193.

#### 3.2.8. (6*S*,12a*S*)-2-(2-(1*H*-Imidazoil)propyl)-6-(4-methoxyphenyl)-2,3,6,7,12,12a-hexaahydropyrazino[1′,2′:1,6]pyrido[3,4-b]indole-1,4-dione (**10**)

The procedure for the synthesis of imidazole **9** was followed with carboxylate **8** (0.50 g, 1.2 mmol), 1-(3-aminopropyl)imidazole (0.3 g, 2.4 mmol), and 15 mL of a DMF/H_2_O mixture (8/2, *v*/*v*) to obtain the product as an off-white solid (0.49 g, 87%). ^1^H NMR (500 MHz, CDCl_3_) δ 8.59 (s, 1H), 7.59 (s, 1H), 7.52 (d, *J* = 7.8 Hz, 1H), 7.31 (d, *J* = 8.0 Hz, 1H), 7.17 (m, 4H), 7.02 (s, 1H), 6.97 (s, 1H), 6.96 (s, 1H), 6.80 (d, *J* = 8.7 Hz, 2H), 4.33 (dd, *J* = 11.9, 4.1 Hz, 1H), 4.07 (d, *J* = 17.0 Hz, 1H), 3.98 (t, *J* = 7 Hz, 2H), 3.90 (d, *J* = 17.6 Hz, 1H) 3.75 (s, 3H), 3.53 (dd, *J* = 15.4, 4.3 Hz, 1H), 3.38 (m, 2H), 2.94 (m, 1H), 2.05 (m, 2H) ppm; ^13^C NMR (126 MHz, CDCl_3_) δ 165.8, 161.4, 160.0, 137.0, 136.4, 130.12, 130.1, 128.6, 126.2, 122.6, 120.0, 118.9, 118.3, 114.2, 111.3, 108.5, 55.2, 52.5, 51.7, 49.6, 44.6, 43.6, 28.1, 27.7 ppm; IR (ATR) ν = 2937, 2841, 1647, 1610, 1509, 1452, 1329, 1244, 1174, 1153, 1032, 842, 738 cm^−1^; ESI-HRMS m/z: calcd for [M+H]^+^ C_27_H_28_N_5_O_3_: 470.2192, found 470.2196.

#### 3.2.9. General Procedure for Synthesis of IMSs

Imidazole **9** or **10** (100 mg, 0.21 mmol) was placed in the MW tube, and alkyl iodide (2 mL) was added. The mixture was placed in an MW reactor and was heated at 110 °C for 5 min. After that time, the reaction mixture was cooled, and n-hexane was added. The mixture was allowed to stand in the ultrasonic bath for 5 min. The precipitated salt was filtered off, and the solid was thoroughly washed with n-hexane to remove any residual unreacted alkyl iodide. After crystallization in CH_2_Cl_2_-diethyl ether, IMSs were isolated by filtration.

IMS **11a**

The general procedure was followed, yielding 92 mg (91% yield) as an off-white solid. ^1^H NMR (500 MHz, DMSO-*d*_6_) δ 11.08 (s, 1H), 9.09 (s, 1H), 7.79 (s, 1H), 7.67 (s, 1H), 7.52 (d, *J* = 7.8 Hz, 1H), 7.32 (d, *J* = 8.1 Hz, 1H), 7.13 (m, 3H), 7.04 (m, 1H), 6.94 (d, *J* = 8.7 Hz, 2H), 6.90 (s, 1H), 4.35 (d, *J* = 17.4 Hz, 1H), 4.16 (t, *J* = 7.1 Hz, 2H), 4.08 (d, *J* = 17.5 Hz, 1H), 3.85 (s, 3H), 3.74 (s, 3H), 3.53 (m, 1H), 3.26 (m, 2H), 3.07 (m, 1H), 2.09 (m, 2H) ppm; ^13^C NMR (126 MHz, DMSO-*d*_6_) δ 165.6, 162.7, 159.7, 137.2, 136.7, 131.5, 131.1, 130.0, 126.4, 124.0, 122.7, 122.1, 119.4, 118.5, 114.6, 111.8, 107.7, 55.7, 52.5, 51.1, 49.3, 47.0, 42.4, 36.2, 27.3, 27.0 ppm; IR (ATR) ν = 2972, 2842, 1738, 1647, 1610, 1509, 1455, 1328, 1246, 1216, 1155, 1027, 839, 741 cm^−1^; ESI-HRMS m/z: calcd for [M-I]^+^ C_28_H_30_N_5_O_3_ 484.2343, found 484.2348.

IMS **11b**

The general procedure was followed, yielding 91 mg (87% yield) as an off-white solid. ^1^H NMR (500 MHz, DMSO-*d*_6_) δ 11.08 (s, 1H), 9.17 (s, 1H), 7.82 (s, 1H), 7.78 (s, 1H), 7.52 (d, *J* = 7.8 Hz, 1H), 7.32 (d, *J* = 8.1 Hz, 1H), 7.13 (m, 3H), 7.04 (m, 1H), 6.94 (d, *J* = 8.7 Hz, 2H), 6.90 (s, 1H), 4.35 (d, *J* = 17.4 Hz, 1H), 4.17 (m, 4H), 4.08 (m, 2H), 3.74 (s, 3H), 3.53 (m, 1H), 3.28 (m, 2H), 3.07 (m, 1H), 2.11 (m, 2H), 1.43 (t, *J* = 7.3 Hz, 3H) ppm; ^13^C NMR (126 MHz, DMSO-*d*_6_) δ 165.6, 162.7, 159.7, 136.7, 136.4, 131.5, 131.1, 130.0, 126.4, 122.9, 122.5, 122.1, 119.4, 118.5, 114.6, 111.8, 107.7, 55.7, 52.5, 51.1, 49.2, 47.0, 44.7, 42.4, 27.3, 26.9, 15.5 ppm; IR (ATR) ν = 2968, 2844, 1739, 1645, 1609, 1509, 1455, 1328, 1245, 1156, 1028, 837, 742 cm^−1^; ESI-HRMS m/z: calcd for [M-I]^+^ C_29_H_32_N_5_O_3_ 498.2500, found 498.2512.

IMS **11c**

The general procedure was followed, yielding 91 mg (85% yield) as an off-white solid. ^1^H NMR (500 MHz, DMSO-*d*_6_) δ 11.08 (s, 1H), 9.16 (s, 1H), 7.83 (s, 2H), 7.78 (s, 1H), 7.52 (d, *J* = 7.8 Hz, 1H), 7.32 (d, *J* = 8.0 Hz, 1H), 7.13 (m, 3H), 7.04 (m, 1H), 6.94 (d, *J* = 8.6 Hz, 2H), 6.90 (s, 1H), 4.35 (d, *J* = 17.5 Hz, 1H), 4.12 (m, 6H), 3.74 (s, 3H), 3.52 (m, 1H), 3.26 (m, 2H), 3.08 (m, 1H), 2.11 (m, 2H), 1.81 (m, 2H), 0.87 (t, *J* = 7.3 Hz, 3H) ppm; ^13^C NMR (126 MHz, DMSO-*d*_6_) δ 165.6, 162.7, 159.7, 136.7, 136.67, 131.5, 131.1, 130.0, 126.4, 122.9, 122.8, 122.1, 119.4, 118.5, 114.6, 111.8, 107.7, 55.7, 52.5, 51.1, 50.8, 49.2, 47.1, 42.3, 27.3, 26.9, 23.3, 10.9 ppm; IR (ATR) ν = 2979, 2838, 1738, 1651, 1509, 1453, 1328, 1247, 1156, 1028, 837, 746 cm^−1^; ESI-HRMS m/z: calcd for [M-I]^+^ C_30_H_34_N_5_O_3_ 512.2656, found 512.2658.

IMS **11d**

The general procedure was followed, yielding 93 mg (84% yield) as an off-white solid. ^1^H NMR (500 MHz, DMSO-*d*_6_) δ 11.08 (s, 1H), 9.17 (s, 1H), 7.83 (s, 1H), 7.77 (s, 1H), 7.52 (d, *J* = 7.8 Hz, 1H), 7.33 (d, *J* = 8.0 Hz, 1H), 7.14 (m, 3H), 7.04 (s, 1H), 6.94 (d, *J* = 8.8 Hz, 2H), 6.90 (s, 1H), 4.35 (d, *J* = 17.4 Hz, 1H), 4.17 (m, 4H), 4.08 (m, 2H), 3.74 (s, 3H), 3.52 (m, 1H), 3.28 (m, 2H), 3.06 (m, 1H), 2.11 (m, 2H), 1.77 (m, 2H), 1.27 (m, 2H), 0.91 (t, *J* = 7.4 Hz, 3H) ppm; ^13^C NMR (126 MHz, DMSO-*d*_6_) δ 165.6, 162.7, 159.7, 136.7, 136.65, 131.5, 131.1, 130.0, 126.4, 122.9, 122.8, 122.1, 119.4, 118.5, 114.6, 111.8, 107.7, 55.7, 52.5, 51.1, 49.2, 49.1, 47.1, 42.4, 31.8, 27.3, 26.9, 19.3, 13.8 ppm; IR (ATR) ν = 2976, 2840, 1738, 1645, 1509, 1455, 1328, 1245, 1155, 1028, 838, 746 cm^−1^; ESI-HRMS m/z: calcd for [M-I]^+^ C_31_H_36_N_5_O_3_ 526.2813, found 526.2824.

IMS **12a**

The general procedure was followed, yielding 91 mg (90% yield) as an off-white solid. ^1^H NMR (500 MHz, DMSO-*d*_6_) δ 11.08 (s, 1H), 9.09 (s, 1H), 7.80 (s, 1H), 7.68 (s, 1H), 7.52 (d, *J* = 7.9 Hz, 1H), 7.32 (d, *J* = 8.1 Hz, 1H), 7.14 (m, 3H), 7.04 (m, 1H), 6.94 (d, *J* = 8.7 Hz, 2H), 6.90 (s, 1H), 4.35 (d, *J* = 17.4 Hz, 1H), 4.16 (t, *J* = 7.1 Hz, 2H), 4.08 (d, *J* = 17.5 Hz, 1H), 3.85 (s, 3H), 3.74 (s, 3H), 3.54 (m, 1H), 3.26 (m, 2H), 3.07 (m, 1H), 2.09 (m, 2H) ppm; ^13^C NMR (126 MHz, DMSO-*d*_6_) δ 165.6, 162.7, 159.7, 137.2, 136.7, 131.5, 131.1, 130.0, 126.4, 124.0, 122.7, 122.1, 119.4, 118.5, 114.6, 111.8, 107.7, 55.7, 52.5, 51.1, 49.3, 47.0, 42.4, 36.2, 27.3, 27.0 ppm; IR (ATR) ν = 2969, 2841, 1738, 1645, 1608, 1509, 1454, 1327, 1245, 1217, 1155, 1031, 840, 743 cm^−1^; ESI-HRMS m/z: calcd for [M-I]^+^ C_28_H_30_N_5_O_3_ 484.2343, found 484.2349.

IMS **12b**

The general procedure was followed, yielding 93 mg (89% yield) as an off-white solid. ^1^H NMR (500 MHz, DMSO-*d*_6_) δ 11.08 (s, 1H), 9.16 (s, 1H), 7.82 (s, 1H), 7.78 (s, 1H), 7.52 (d, *J* = 7.8 Hz, 1H), 7.32 (d, *J* = 8.1 Hz, 1H), 7.14 (m, 3H), 7.05 (m, 1H), 6.95 (d, *J* = 8.7 Hz, 2H), 6.90 (s, 1H), 4.35 (d, *J* = 17.4 Hz, 1H), 4.17 (m, 4H), 4.08 (m, 2H), 3.74 (s, 3H), 3.53 (m, 1H), 3.26 (m, 2H), 3.07 (m, 1H), 2.11 (m, 2H), 1.43 (t, *J* = 7.3 Hz, 3H) ppm; ^13^C NMR (126 MHz, DMSO-*d*_6_) δ 165.6, 162.7, 159.7, 136.7, 136.4, 131.5, 131.1, 130.0, 126.4, 122.9, 122.5, 122.1, 119.4, 118.5, 114.6, 111.8, 107.7, 55.7, 52.5, 51.1, 49.2, 47.1, 44.7, 42.4, 27.3, 26.9, 15.5 ppm; IR (ATR) ν = 2972, 2842, 1738, 1645, 1608, 1509, 1455, 1328, 1246, 1156, 1027, 836, 745 cm^−1^; ESI-HRMS m/z: calcd for [M-I]^+^ C_29_H_32_N_5_O_3_ 498.2500, found 498.2507.

IMS **12c**

The general procedure was followed, yielding 91 mg (87% yield) as an off-white solid. ^1^H NMR (500 MHz, DMSO-*d*_6_) δ 11.08 (s, 1H), 9.16 (s, 1H), 7.83 (s, 2H), 7.77 (s, 1H), 7.51 (d, *J* = 7.8 Hz, 1H), 7.32 (d, *J* = 8.0 Hz, 1H), 7.14 (m, 3H), 7.04 (m, 1H), 6.94 (d, *J* = 8.6 Hz, 2H), 6.90 (s, 1H), 4.35 (d, *J* = 17.5 Hz, 1H), 4.12 (m, 6H), 3.74 (s, 3H), 3.53 (m, 1H), 3.28 (m, 2H), 3.07 (m, 1H), 2.11 (m, 2H), 1.81 (m, 2H), 0.87 (t, *J* = 7.3 Hz, 3H) ppm; ^13^C NMR (126 MHz, DMSO-*d*_6_) δ 165.6, 162.7, 159.7, 136.7, 136.67, 131.5, 131.1, 130.0, 126.4, 122.9, 122.8, 122.1, 119.4, 118.5, 114.6, 111.8, 107.7, 55.7, 52.5, 51.1, 50.8, 49.2, 47.1, 42.3, 27.3, 26.9, 23.3, 10.9 ppm; IR (ATR) ν = 2977, 2839, 1740, 1649, 1508, 1457, 1329, 1247, 1155, 1027, 837, 745 cm^−1^; ESI-HRMS m/z: calcd for [M-I]^+^ C_30_H_34_N_5_O_3_ 512.2656, found 512.2654.

IMS **12d**

The general procedure was followed, yielding 94 mg (85% yield) as an off-white solid. ^1^H NMR (500 MHz, DMSO-*d*_6_) δ 11.08 (s, 1H), 9.17 (s, 1H), 7.83 (s, 1H), 7.77 (s, 1H), 7.52 (d, *J* = 7.8 Hz, 1H), 7.33 (d, *J* = 8.0 Hz, 1H), 7.13 (m, 3H), 7.04 (s, 1H), 6.94 (d, *J* = 8.8 Hz, 2H), 6.90 (s, 1H), 4.35 (d, *J* = 17.4 Hz, 1H), 4.18 (m, 4H), 4.07 (m, 2H), 3.74 (s, 3H), 3.54 (m, 1H), 3.28 (m, 2H), 3.07 (m, 1H), 2.11 (m, 2H), 1.77 (m, 2H), 1.27 (m, 2H), 0.92 (t, *J* = 7.4 Hz, 3H) ppm; ^13^C NMR (126 MHz, DMSO-*d*_6_) δ 165.6, 162.7, 159.7, 136.7, 136.64, 131.5, 131.1, 130.0, 126.4, 122.9, 122.8, 122.1, 119.4, 118.5, 114.6, 111.8, 107.7, 55.7, 52.5, 51.1, 49.2, 49.1, 47.1, 42.4, 31.8, 27.3, 26.9, 19.3, 13.8 ppm; IR (ATR) ν = 2977, 2841, 1738, 1646, 1509, 1455, 1329, 1245, 1156, 1027, 840, 745 cm^−1^; ESI-HRMS m/z: calcd for [M-I]^+^ C_31_H_36_N_5_O_3_ 526.2813, found 526.2817.

### 3.3. Enzyme Inhibition Assays

The inhibition effects of new IMS (**11a**–**d**, **12a**–**d**) and tacrine on AChE from electric eel (*Electrophorus electricus*) and BChE from equine serum were determined according to the Ellman method [47]. The tested compounds were dissolved in DMSO and subsequently diluted with 100 mM Tris buffer to concentrations of 0.01–10 mM, corresponding to final well concentrations of 0.25 μM, 2.5 μM, 25 μM, and 250 μM. Acetylthiocholine iodide and butyrylthiocholine iodide were used as substrates for AChE and BChE, respectively. The experimental procedures for cholinesterase activity assays were performed according to Sigma-Aldrich technical bulletin MAK324. Enzymatic hydrolysis of these substrates leads to the formation of thiocholine, which subsequently reacts with DTNB to produce the yellow-colored 5-thio-2-nitrobenzoate anion. The absorbance of this product was measured spectrophotometrically at 412 nm. Measurements were performed using an Infinite M200 fluorescence spectrophotometer (TECAN, Männedorf, Switzerland) in duplicate. Each assay included two control wells: a blank (no enzyme) and a reference control containing AChE without inhibitor. AChE activity was calculated as a percentage inhibition relative to the control.

### 3.4. Computational Method

ADMET properties were predicted using the pkCSM web server, a computational tool for the in silico assessment of pharmacokinetic and toxicity profiles based on graph-based signatures of small molecules (https://biosig.lab.uq.edu.au/pkcsm/, accessed on 16 April 2026).

### 3.5. Molecular Docking Studies

Molecular docking studies were performed to investigate the binding mode of compound **12c** toward AChE. The enzyme structure (PDB ID: 4EY7) was used as the target protein after standard preparation, including removal of water molecules and addition of hydrogen atoms. Docking calculations were carried out using the SwissDock server with the Attracting Cavities 2.0 method [53,54]. The docking procedure was performed using a blind docking approach, and the resulting poses were clustered and ranked according to estimated binding free energy values. To further validate the predicted binding site, cavity detection and docking were additionally performed using CB-Dock2. The predicted binding cavities were ranked based on docking scores, and contact residues were analyzed to identify key ligand-protein interactions.

## 4. Conclusions

In this study, a novel class of hybrid THβC-IMS incorporating a fused diketopiperazine scaffold was successfully designed, synthesized, and evaluated as cholinesterase inhibitors relevant to Alzheimer’s disease. The obtained results demonstrate that molecular hybridization of the THβC core with a cationic IM moiety constitutes an effective strategy for the development of bioactive compounds targeting cholinesterases. All synthesized derivatives exhibited micromolar inhibitory activity toward AChE and BChE, with a clear preference for AChE. Structure–activity relationship analysis revealed that both stereochemistry and alkyl chain length are critical determinants of biological activity. In particular, *S*,*S*-configured derivatives consistently showed higher potency than their *R*,*R* counterparts, highlighting the importance of spatial arrangement for optimal binding within the enzyme active site. Furthermore, elongation of the *N*-alkyl substituent enhanced inhibitory activity, likely due to increased hydrophobic interactions within the catalytic gorge. Among the investigated compounds, derivative **12c** emerged as the most promising candidate, combining high AChE inhibitory potency, favorable selectivity, and an improved predicted safety profile compared to other analogs. ADMET analysis indicated acceptable physicochemical properties and CNS permeability, although potential hepatotoxicity and mutagenicity for selected compounds highlight the need for further structural optimization. Moreover, the in silico results suggest that compound **12c** may act as a potential dual-site AChE inhibitor by simultaneously interacting with both the PAS and CAS residues within the enzyme gorge. Overall, the presented results identify THβC-IM hybrids as a promising new scaffold for developing cholinesterase inhibitors. Future studies will focus on detailed mechanistic investigations, including molecular docking and enzyme kinetics, as well as the evaluation of additional biological activities relevant to Alzheimer’s disease, such as anti-amyloid and neuroprotective effects. Further structural refinement will aim to improve safety profiles while maintaining high inhibitory potency.

## Figures and Tables

**Table 1:** IMSs based on THβC core and yield of MW reactions.

IMS	R	Yield
**11a**	CH_3_	91%
**11b**	C_2_H_5_	87%
**11c**	C_3_H_7_	85%
**11d**	C_4_H_9_	84%
**12a**	CH_3_	90%
**12b**	C_2_H_5_	89%
**12c**	C_3_H_7_	87%
**12d**	C_4_H_9_	85%

**Table 2:** In vitro ChE inhibitory activities of THβC-IMSs.

IMS	Configuration	*N*-Substituent	IC_50_ [μM] for AChE	IC_50_ [μM] for BChE	SI ^a^
**11a**	*R*,*R*	methyl	4.41 ± 0.24	27.12 ± 0.11	6.2
**11b**	*R*,*R*	ethyl	4.22 ± 0.26	22.37 ± 0.23	5.3
**11c**	*R*,*R*	propyl	3.51 ± 0.17	17.71 ± 0.14	5.1
**11d**	*R*,*R*	butyl	2.39 ± 0.22	4.93 ± 0.15	2.0
**12a**	*S*,*S*	methyl	2.92 ± 0.27	17.22 ± 0.22	5.9
**12b**	*S*,*S*	ethyl	2.48 ± 0.28	13.44 ± 0.11	5.4
**12c**	*S*,*S*	propyl	1.24 ± 0.15	10.12 ± 0.34	8.4
**12d**	*S*,*S*	butyl	0.72 ± 0.17	5.23 ± 0.15	7.4
**TAC**			0.07 ± 0.01	0.08 ± 0.01	1.1

^a^ SI = selectivity index; SI = $\frac{{IC}_{50}BChE}{{IC}_{50}AChE}$.

**Table 3:** Selected ADMET properties of THβC-based IMSs.

	THβC-Based IMSs			
ADMET	11a, 12a	11b, 12b	11c, 12c	11d, 12d
molar mass	484.58	498.61	512.63	526.66
logP	2.58	3.06	3.45	3.84
water solubility	moderate	moderate	moderate	moderate
BBB permeability	moderate	moderate	moderate	moderate
CNS permeability	moderate	moderate	moderate	moderate
mutagenicity	yes	no	no	yes
hepatotoxicity	yes	yes	yes	yes
oral rat acute toxicity (LD_50_)	2.55 mol/kg	2.55 mol/kg	2.51 mol/kg	2.48 mol/kg
max. tolerated dose (human)	1.38 mg/kg/day	1.36 mg/kg/day	1.39 mg/kg/day	1.37 mg/kg/day
	2.9 μmol/kg/day	2.7 μmol/kg/day	2.7 μmol/kg/day	2.6 μmol/kg/day

## Data Availability

The data are available on request from the corresponding author.

## Supplementary Materials

The following supporting information can be downloaded at: https://www.mdpi.com/article/10.3390/molecules31101563/s1, NMR spectra of all new compounds.

## Abbreviations

The following abbreviations are used in this manuscript:

AChE	acetylcholinesterase
AD	Alzheimer’s disease
Aβ	amyloid-β
BBB	blood–brain barrier
BChE	butyrylcholinesterase
CAS	catalytic active site
CNS	central nervous system
ChE	cholinesterase
DCM	dichloromethane
DMF	dimethylformamide
DMSO	dimethylsulfoxide
DTNB	5,5′-dithiobis-2-nitrobenzoic acid
IM	imidazole
IMS	imidazolium salt
MW	microwave
PAS	peripheral anionic site
PDE5	phosphodiesterase type 5
THβC	tetrahydro-β-carboline
SAR	structure–activity relationship
SI	selectivity index
