# Supplementary material for: Design, Synthesis, and Biological Evaluation of Highly Functionalized Tetrahydro-β-carboline-imidazolium Hybrids Targeting Cholinesterases
Source: Molecules. 2026 May 8;31(10):1563. doi: 10.3390/molecules31101563 (PMC13209494; doi:10.3390/molecules31101563)

Supplementary Materials

Design, Synthesis, and Biological Evaluation of Highly Functionalized Tetrahydro-β-carboline-imidazolium Hybrids Targeting Cholinesterases

Agnieszka Hryniewicka*, Damian Pawelski and Marta Eliza Plonska-Brzezinska

Department of Organic Chemistry, Faculty of Medicine, Division of Dentistry and Division of Medical Education in English, Medical University of Bialystok, Mickiewicza 2A, 15-222 Bialystok, Poland
* Correspondence: agnieszka.hryniewicka@umb.edu.pl

NMR spectra of new compounds

1. NMR spectra of compound **7** 3
2. NMR spectra of compound **8** 4
3. NMR spectra of imidazole **9** 5
4. NMR spectra of imidazole **10** 6
5. NMR spectra of imidazolium salt **11a** 7
6. NMR spectra of imidazolium salt **11b** 8
7. NMR spectra of imidazolium salt **11c** 9
8. NMR spectra of imidazolium salt **11d** 10
9. NMR spectra of imidazolium salt **12a** 11
10. NMR spectra of imidazolium salt **12b** 12
11. NMR spectra of imidazolium salt **12c** 13
12. NMR spectra of imidazolium salt **12d** 14

1. NMR spectra of compound **7**

^{1}H NMR spectrum of **7**

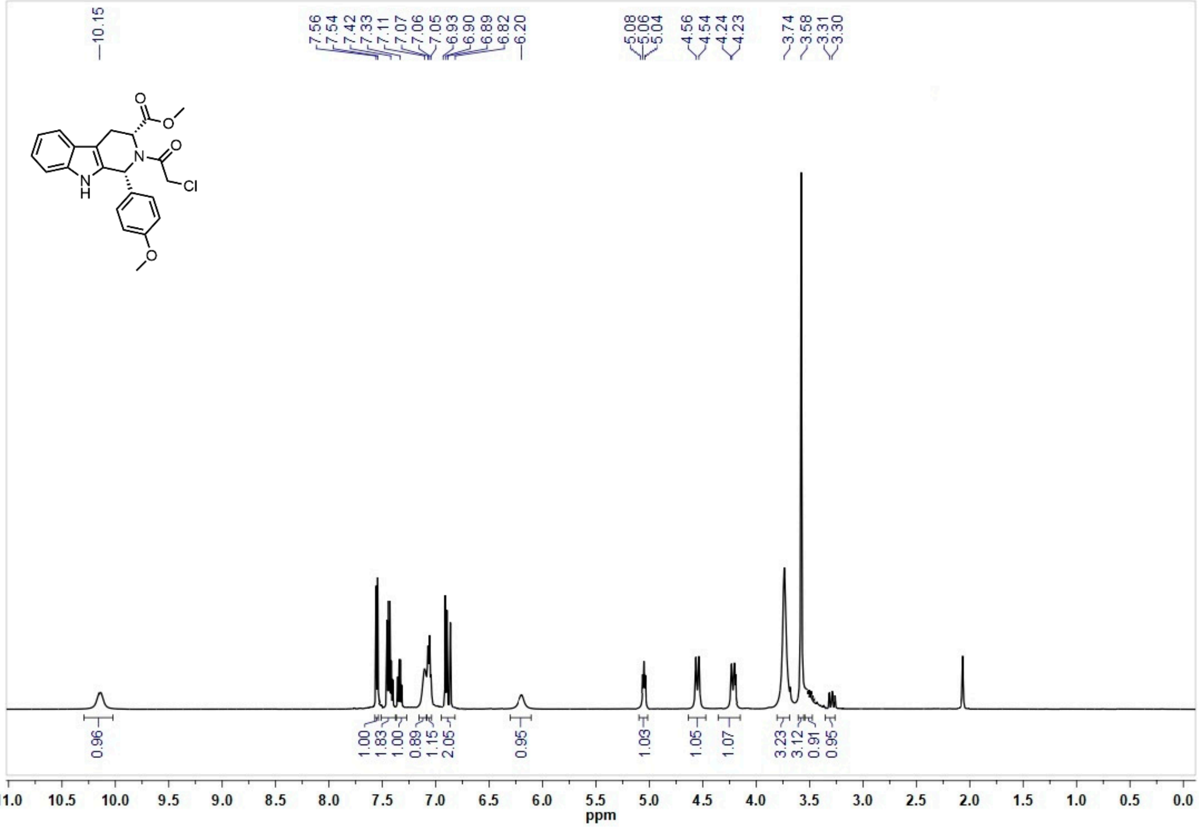

^{13}C NMR spectrum of **7**

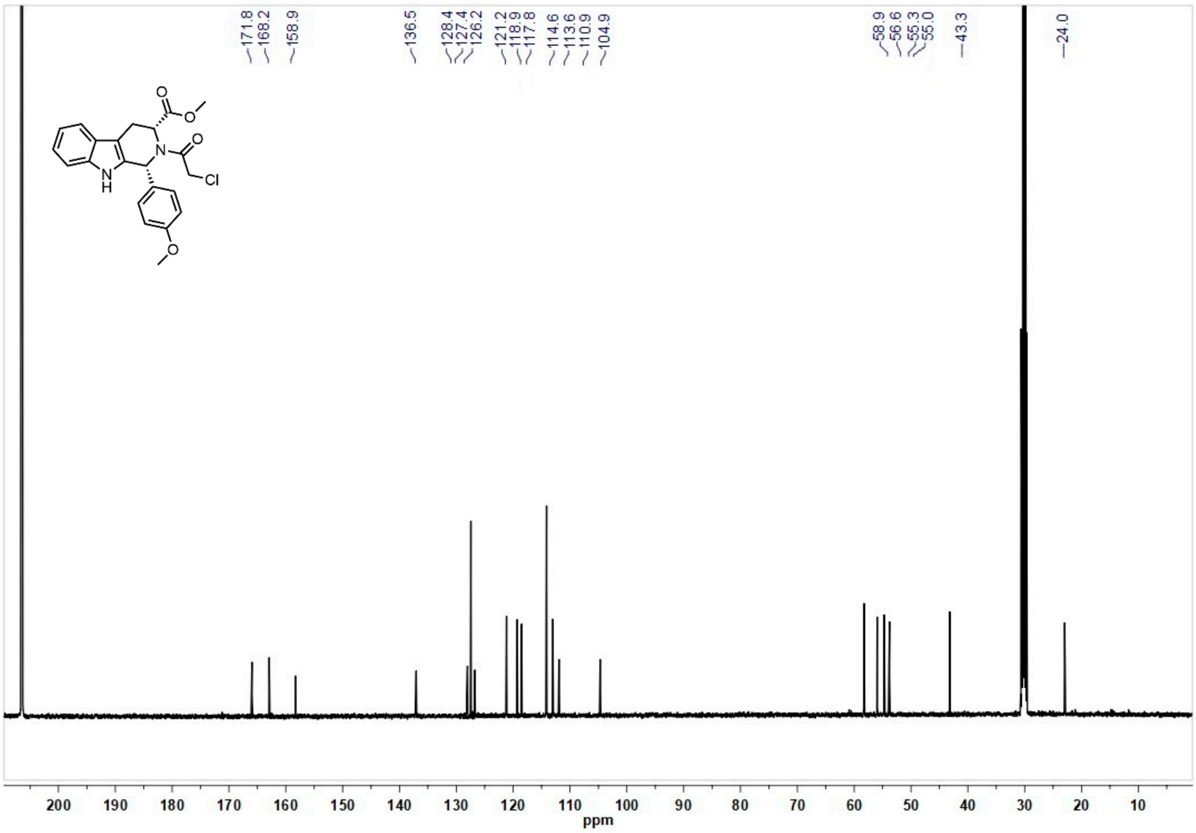

2. NMR spectra of compound **8**

^{1}H NMR spectrum of **8**

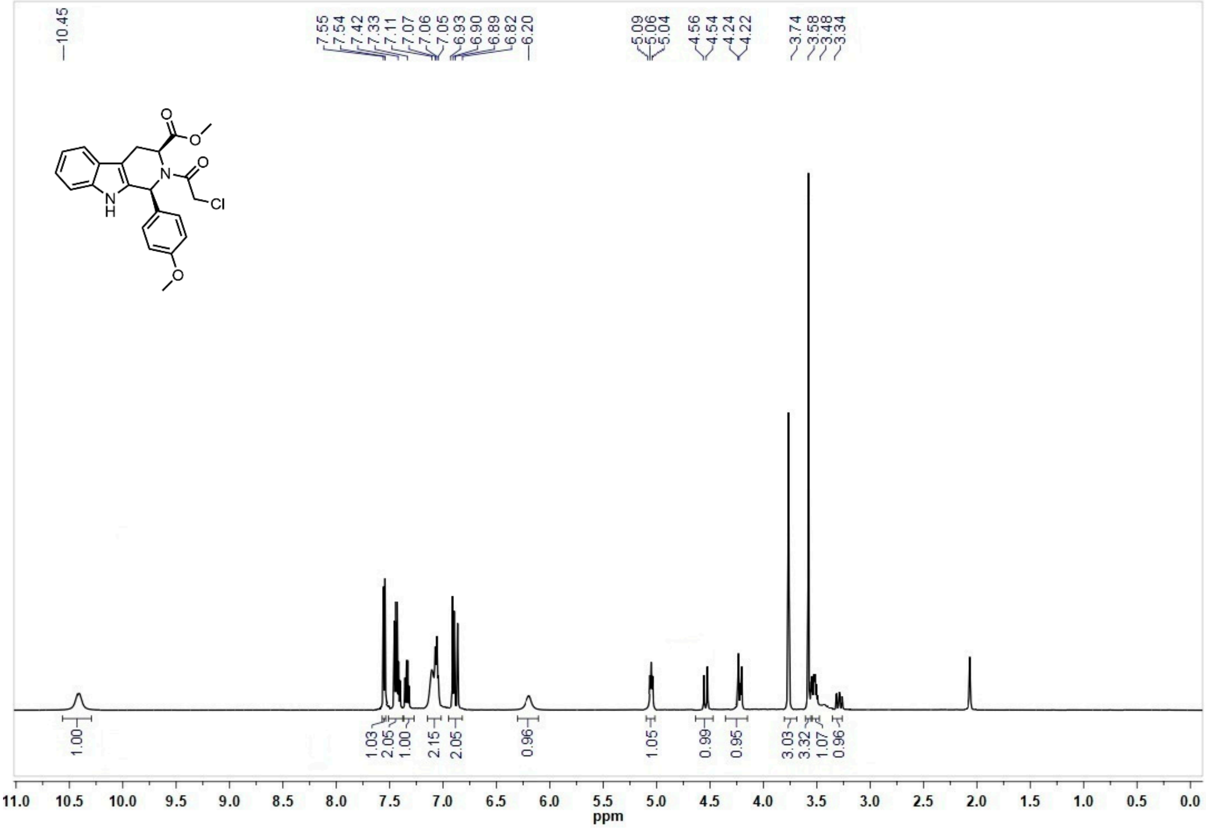

^{13}C NMR spectrum of **8**

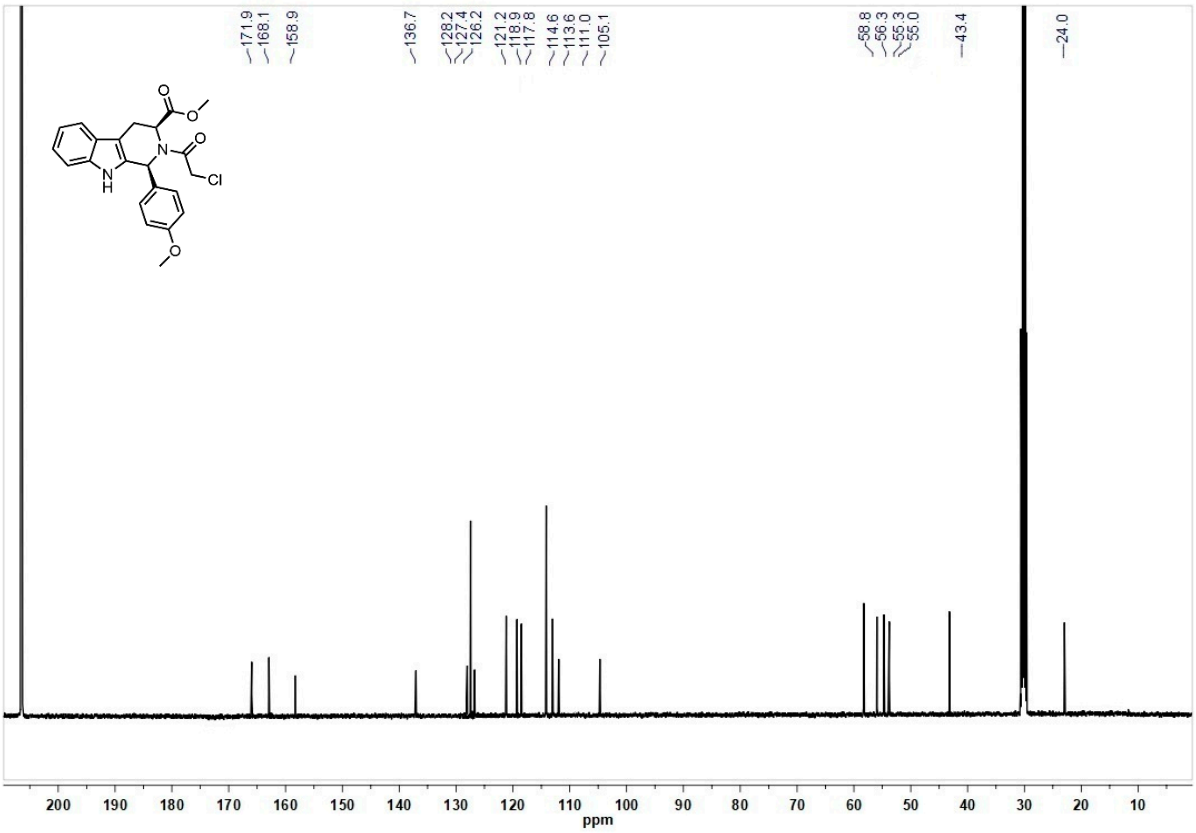

3. NMR spectra of imidazole **9**

^{1}H NMR spectrum of **9**

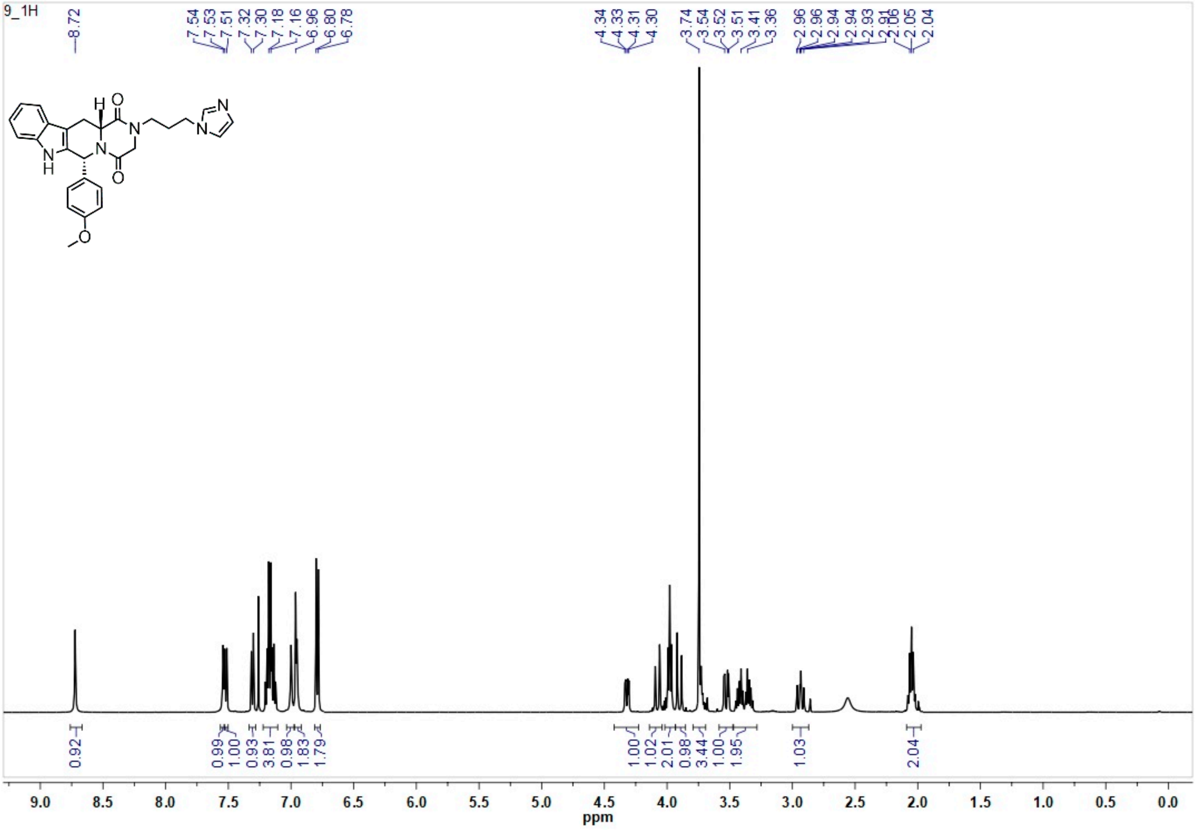

^{13}C NMR spectrum of **9**

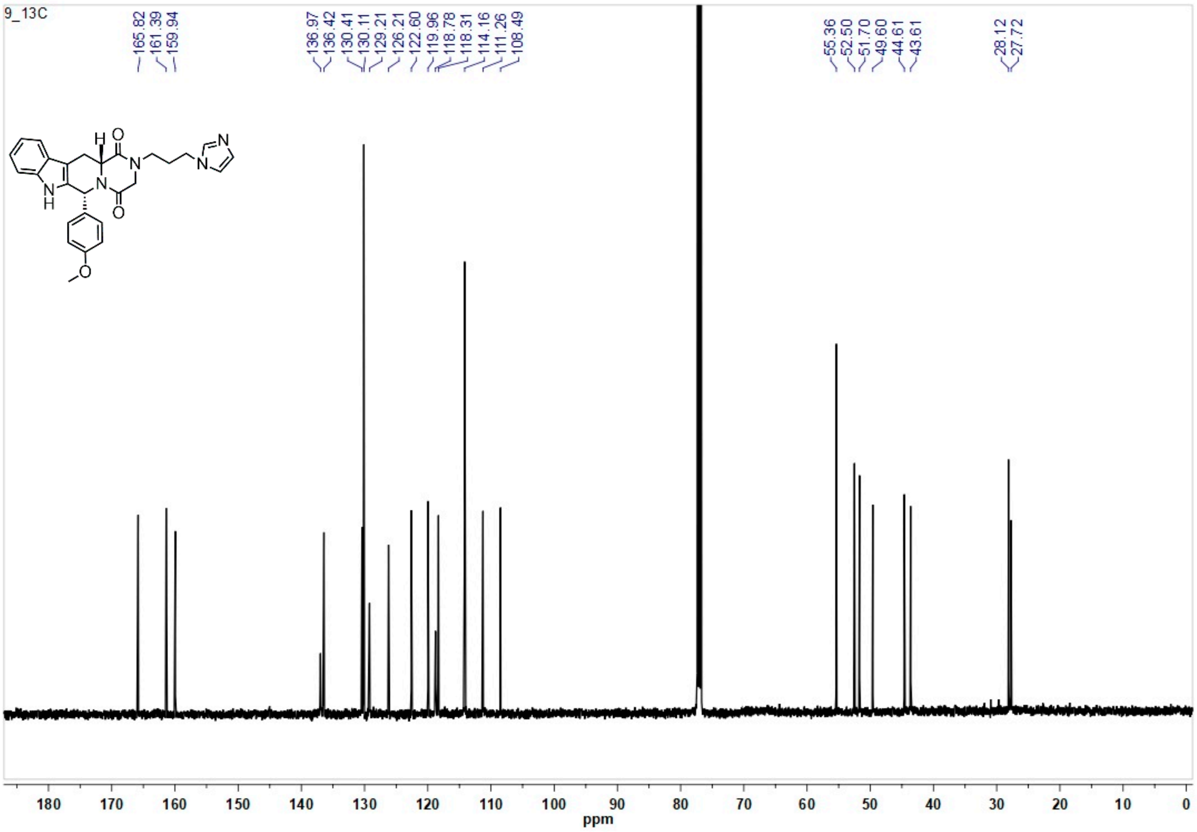

4. NMR spectra of imidazole **10**

^{1}H NMR spectrum of **10**

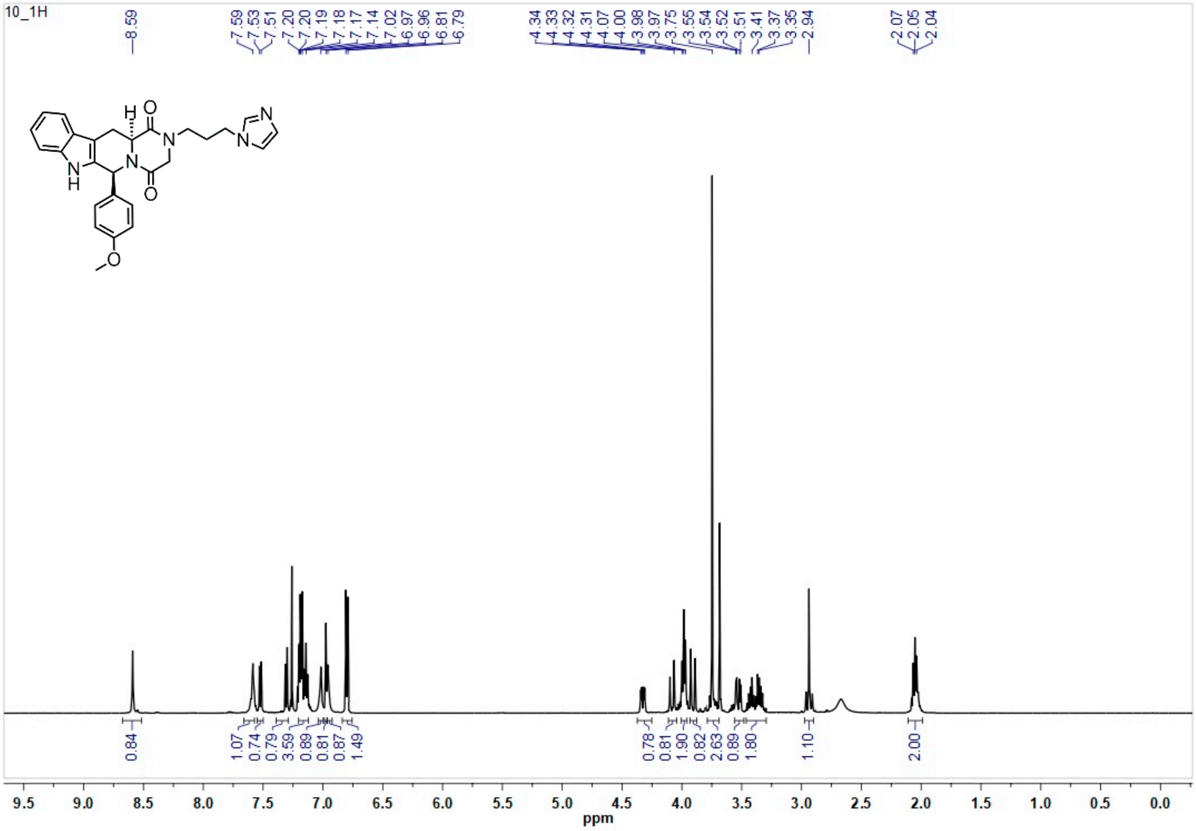

^{13}C NMR spectrum of **10**

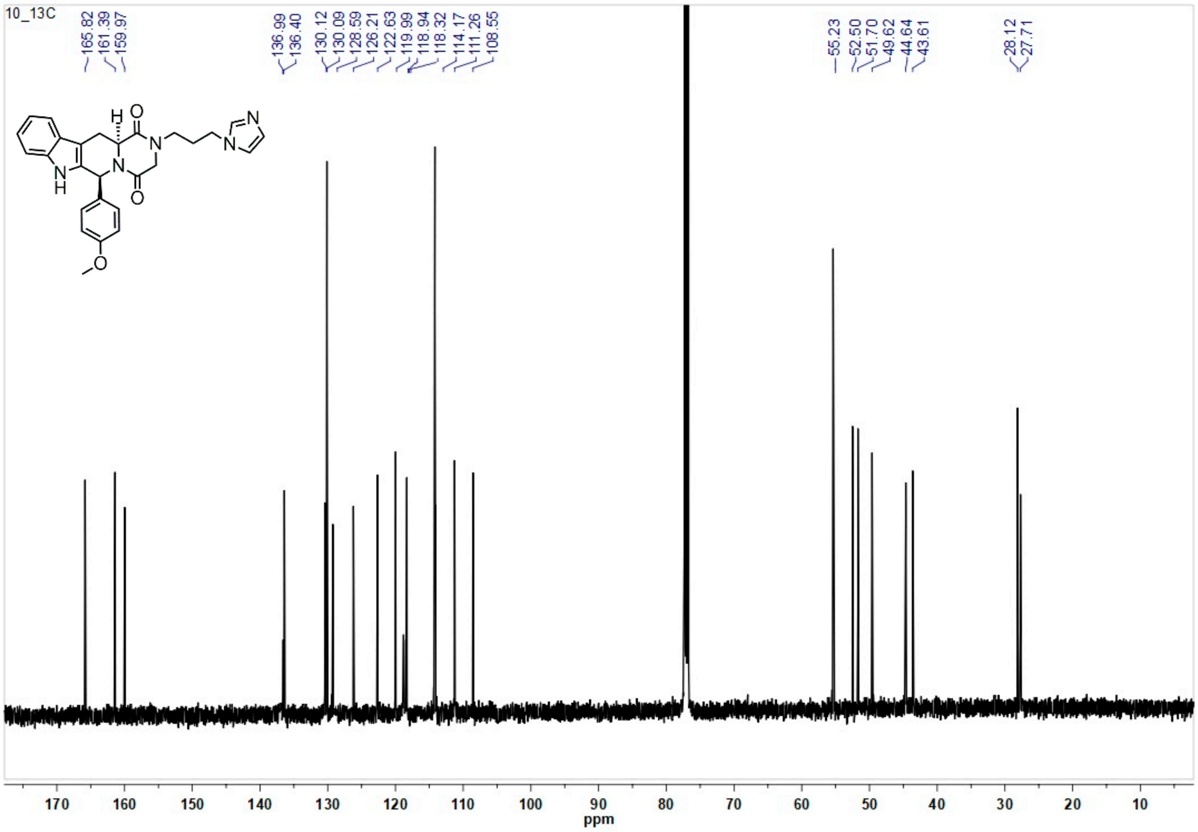

5. NMR spectra of imidazolium salt **11a**

^{1}H NMR spectrum of **11a**

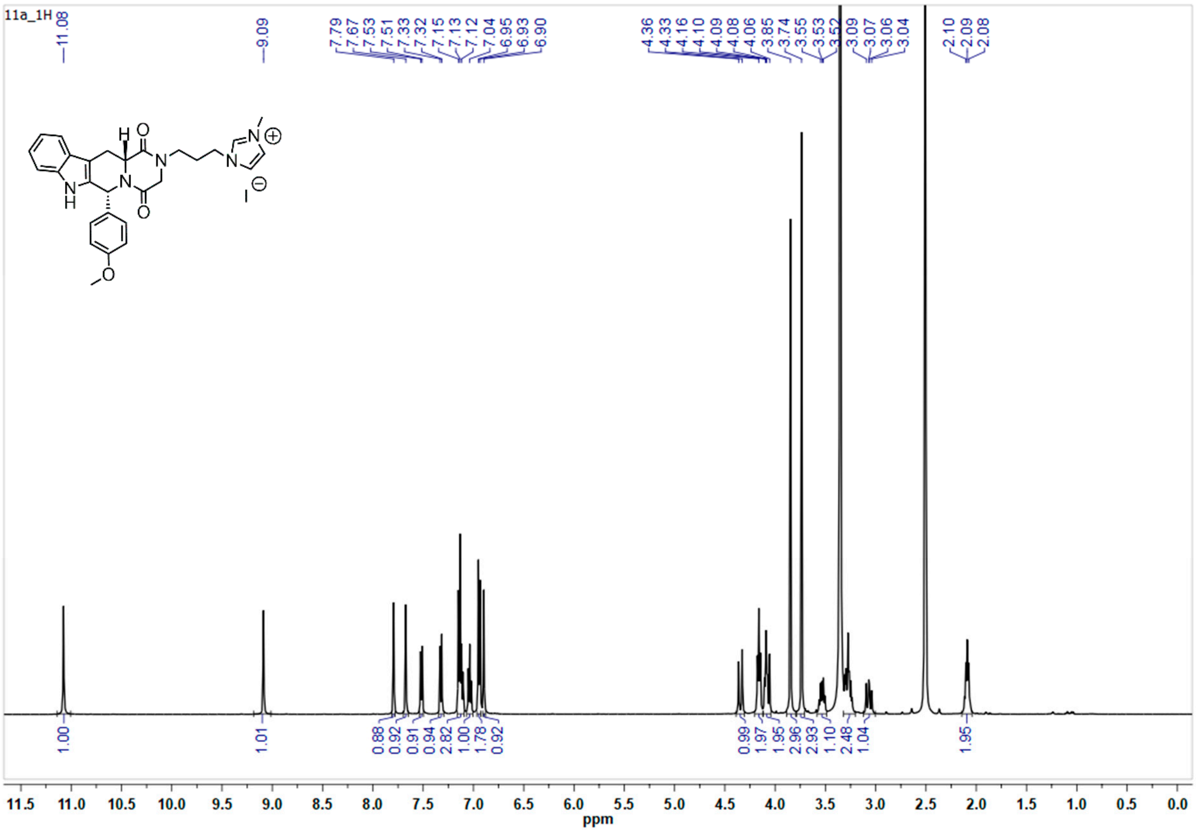

^{13}C NMR spectrum of **11a**

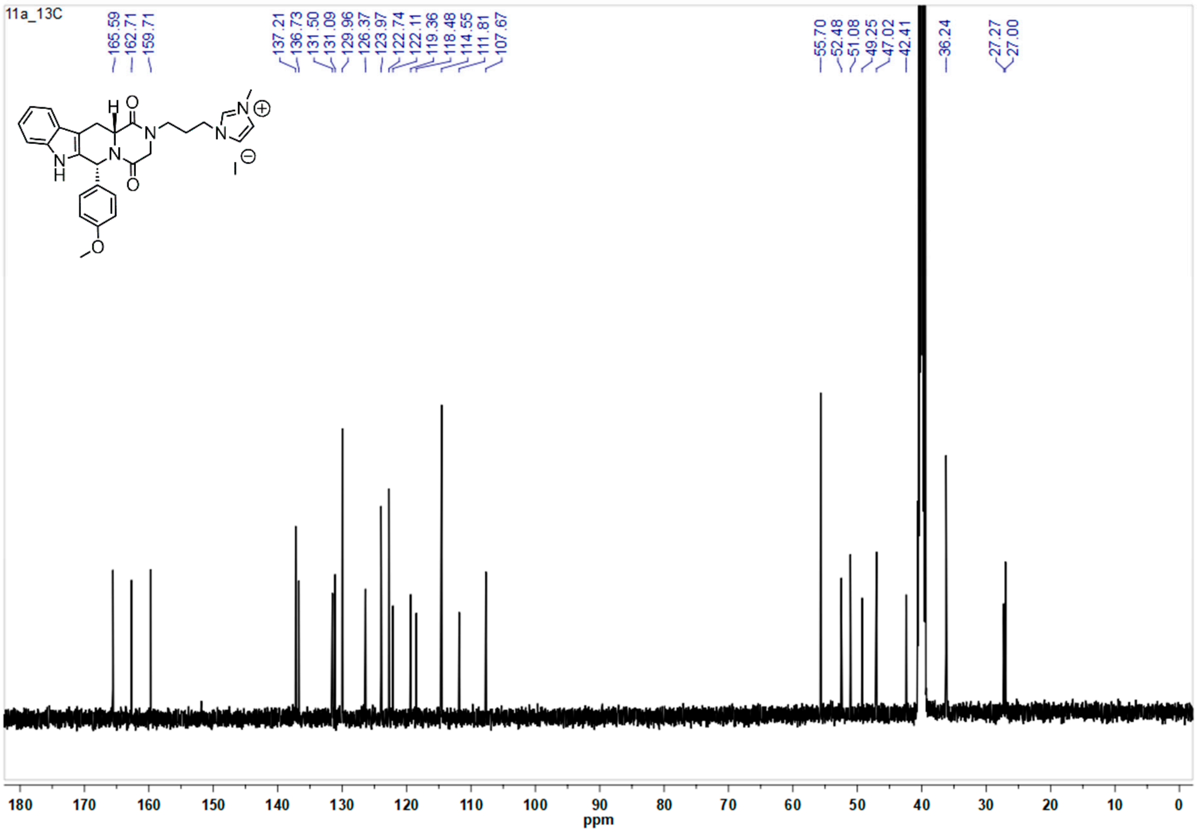

6. NMR spectra of imidazolium salt **11b**

^{1}H NMR spectrum of **11b**

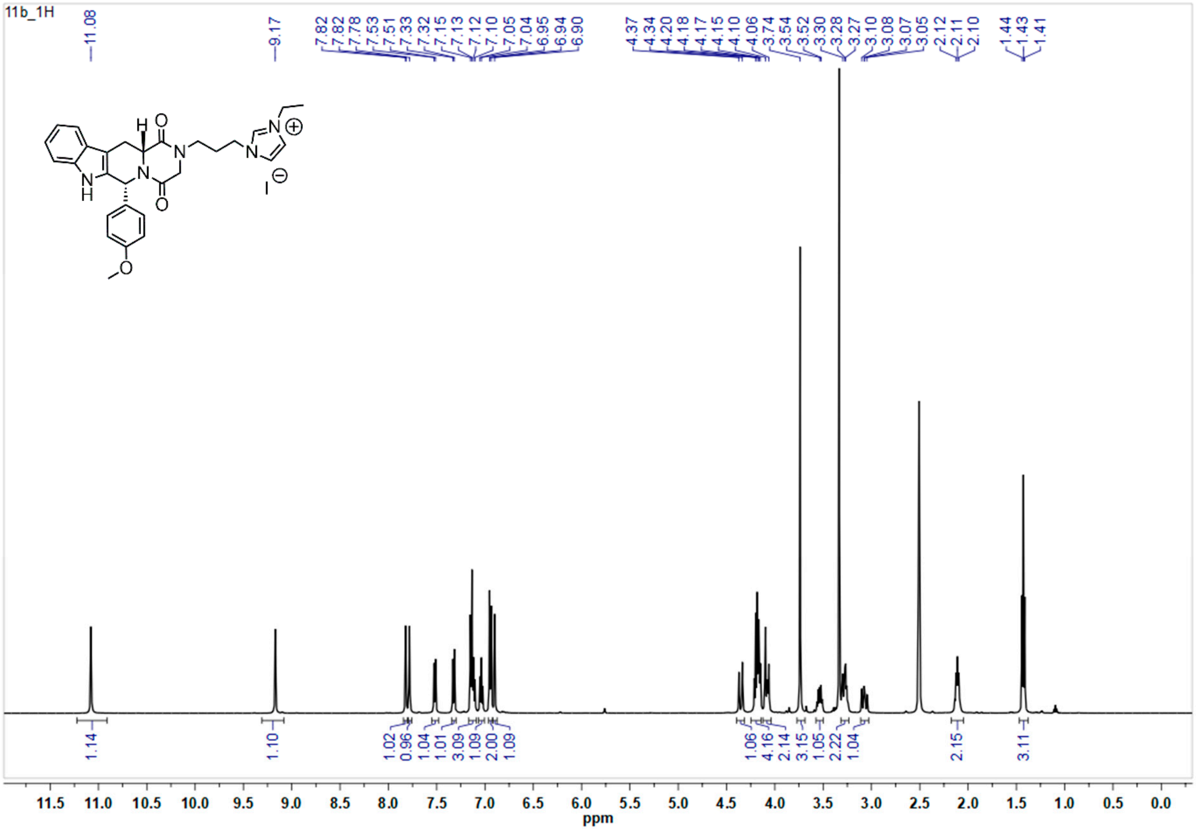

^{13}C NMR spectrum of **11b**

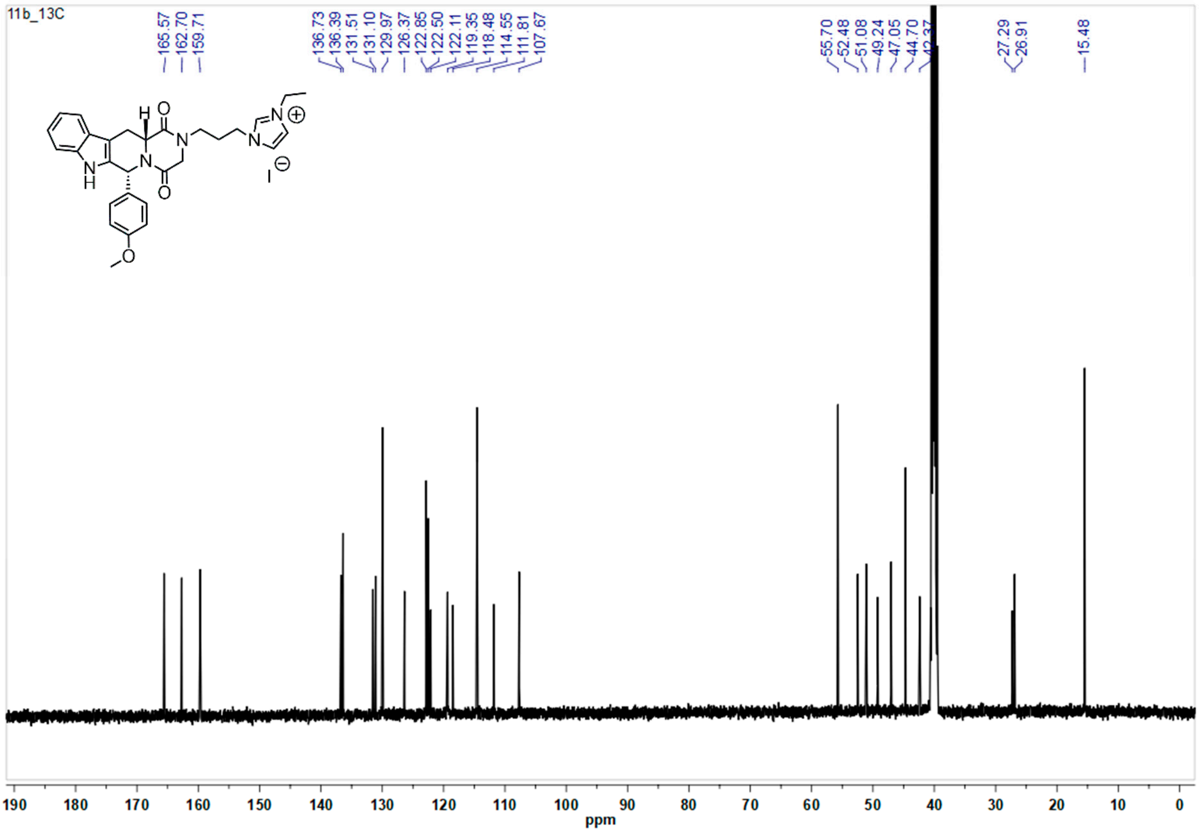

7. NMR spectra of imidazolium salt **11c**

^{1}H NMR spectrum of **11c**

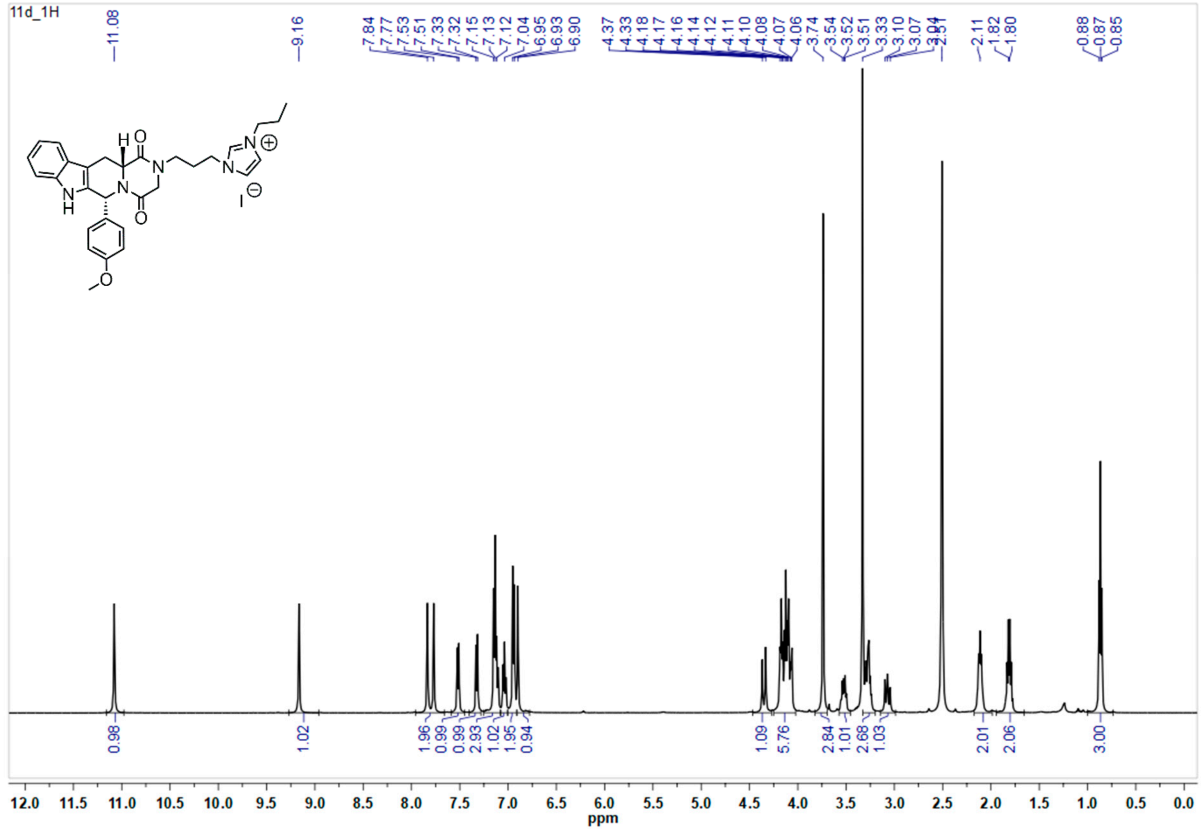

^{13}C NMR spectrum of **11c**

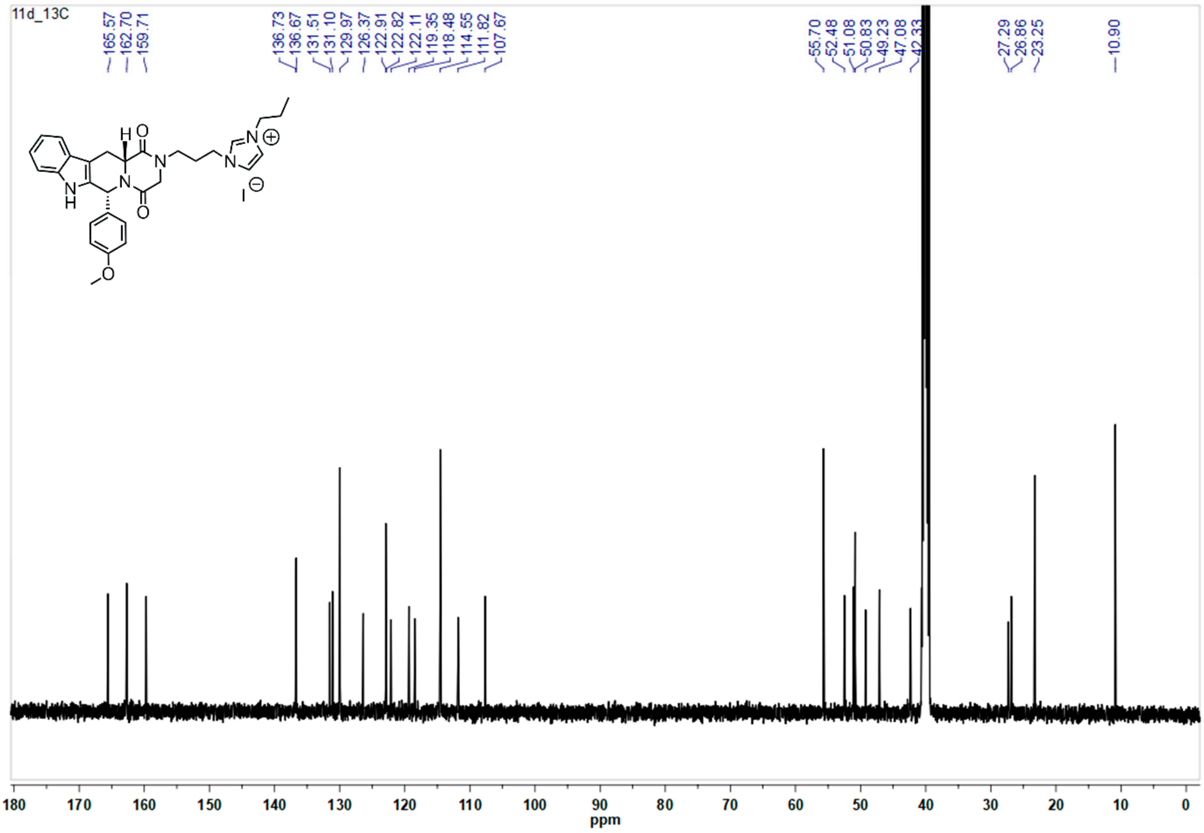

8. NMR spectra of imidazolium salt **11d**

^{1}H NMR spectrum of **11d**

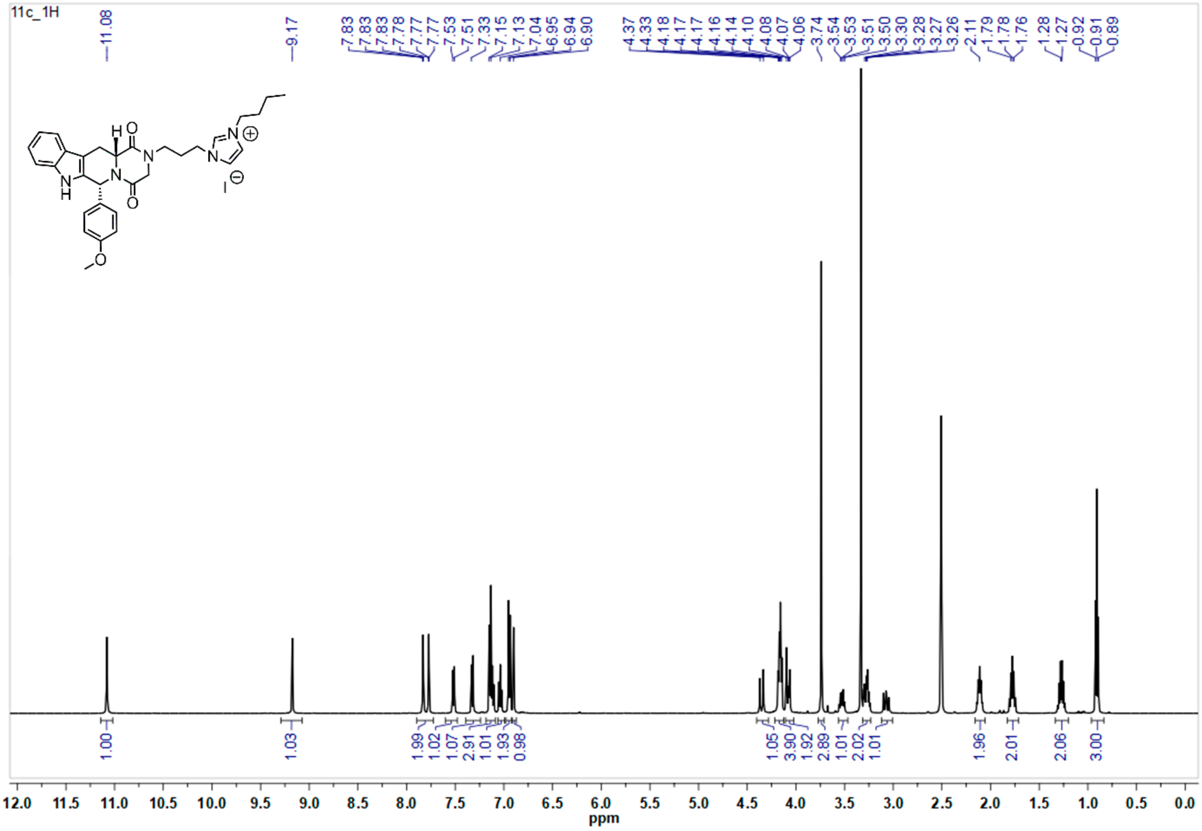

^{13}C NMR spectrum of **11d**

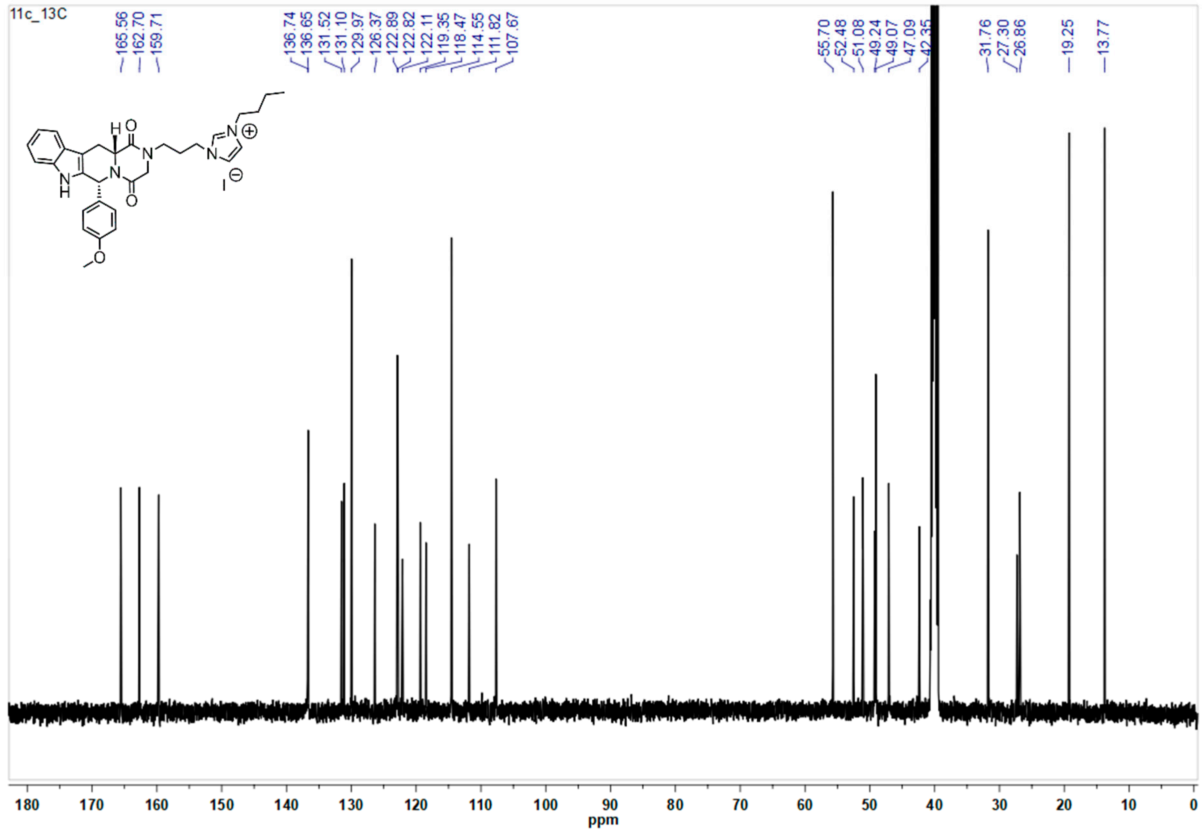

9. NMR spectra of imidazolium salt **12a**

^{1}H NMR spectrum of **12a**

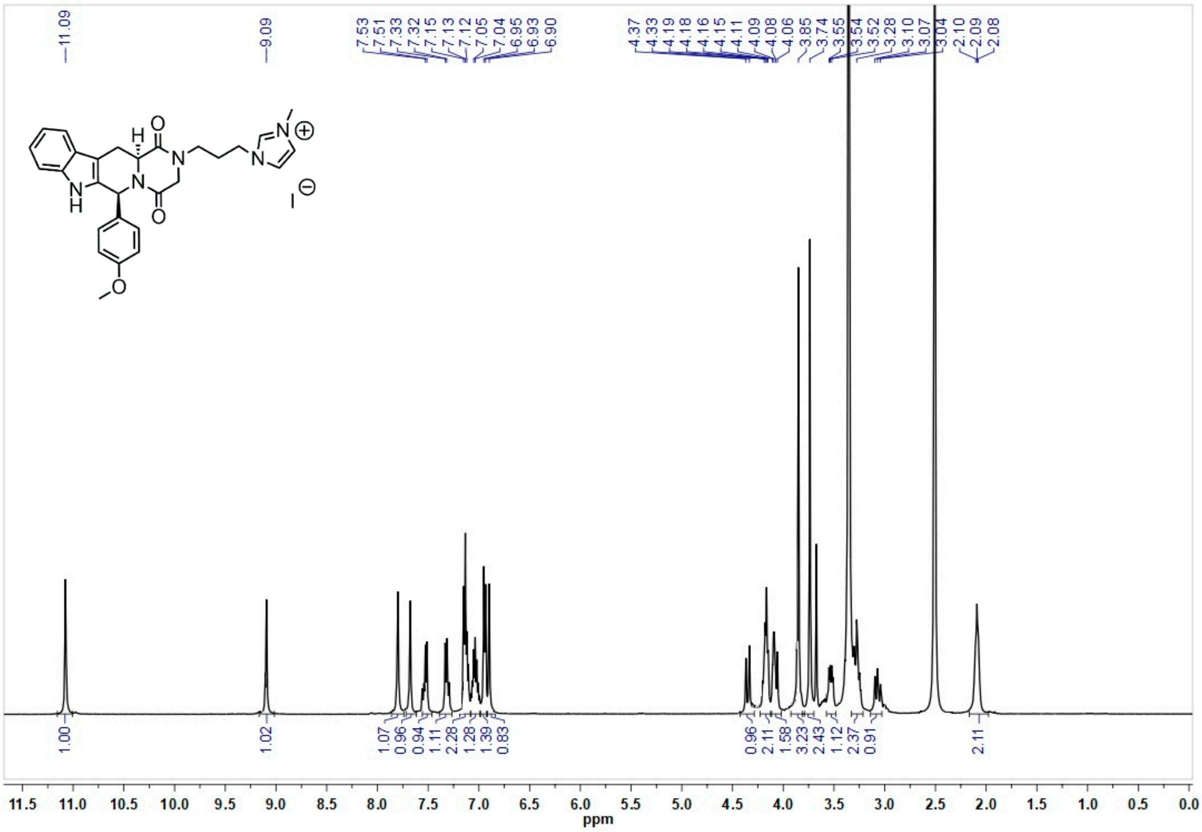

^{13}C NMR spectrum of **12a**

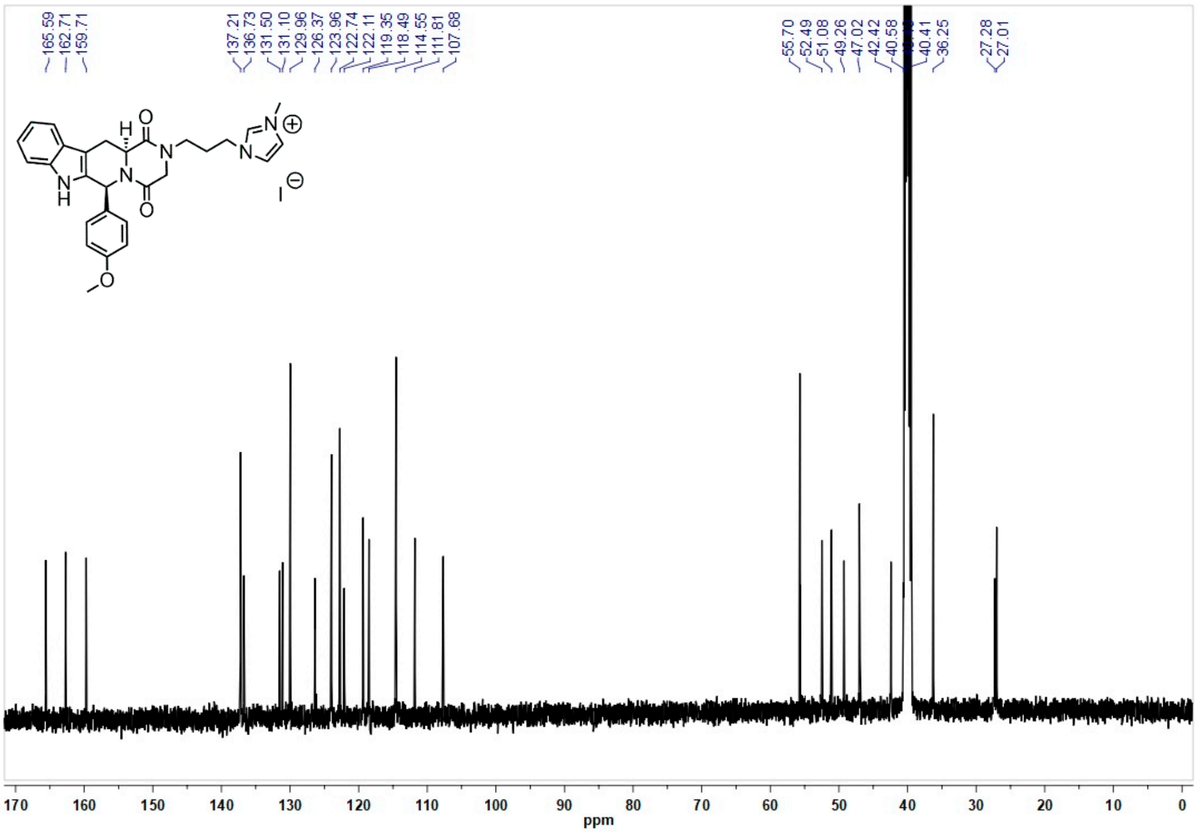

10. NMR spectra of imidazolium salt **12b**

^{1}H NMR spectrum of **12b**

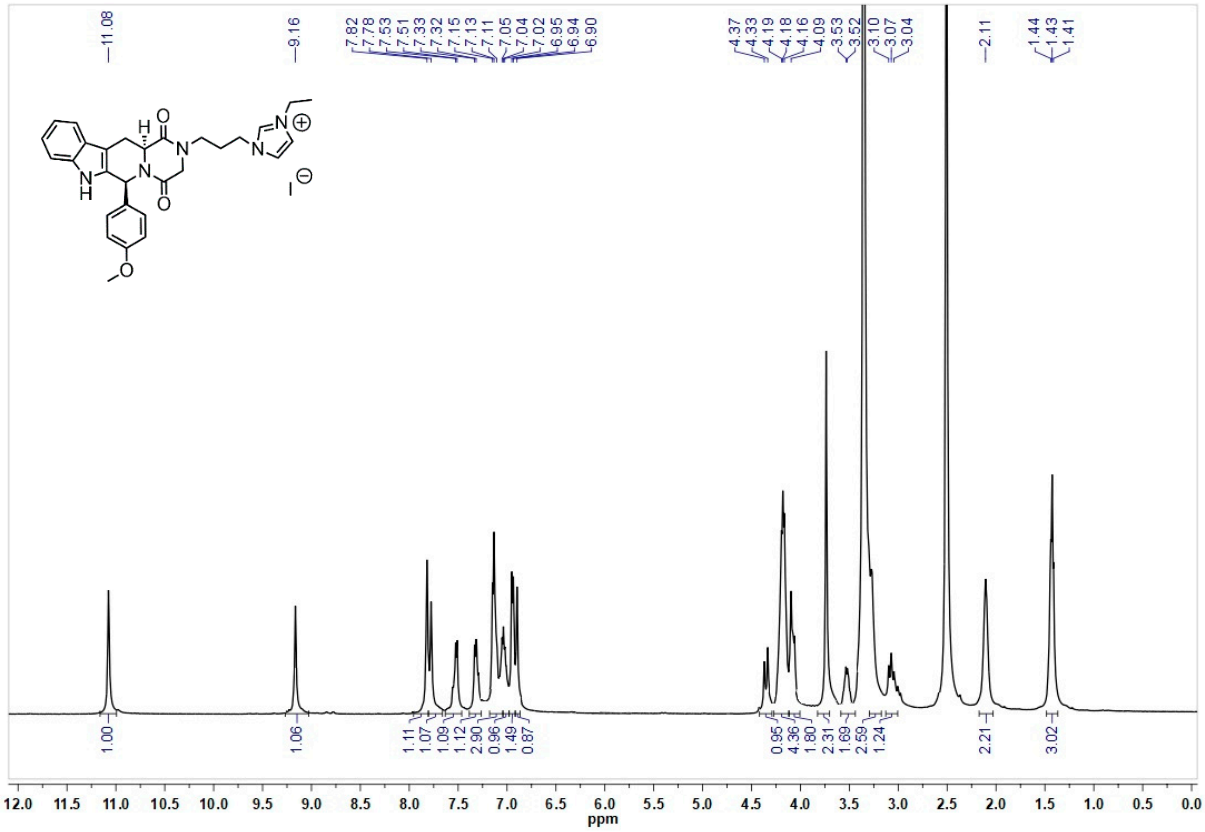

^{13}C NMR spectrum of **12b**

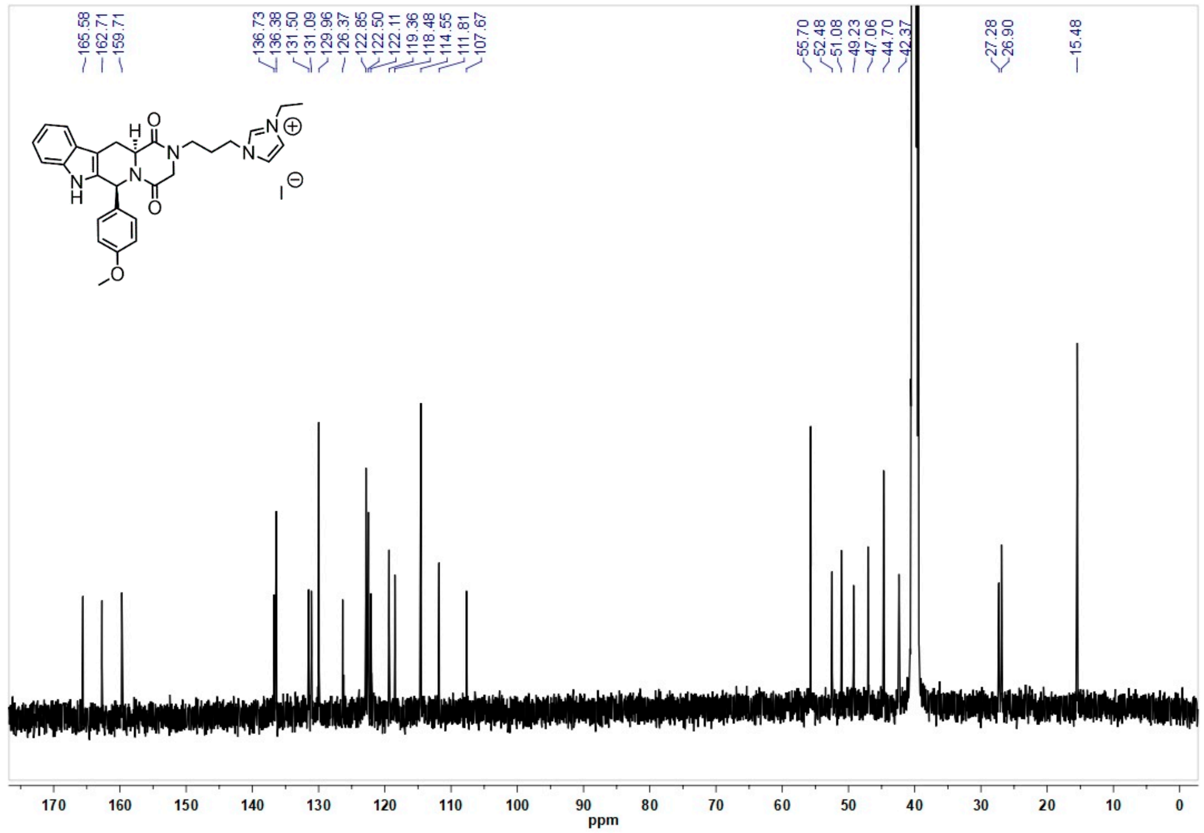

11. NMR spectra of imidazolium salt **12c**

^{1}H NMR spectrum of **12c**

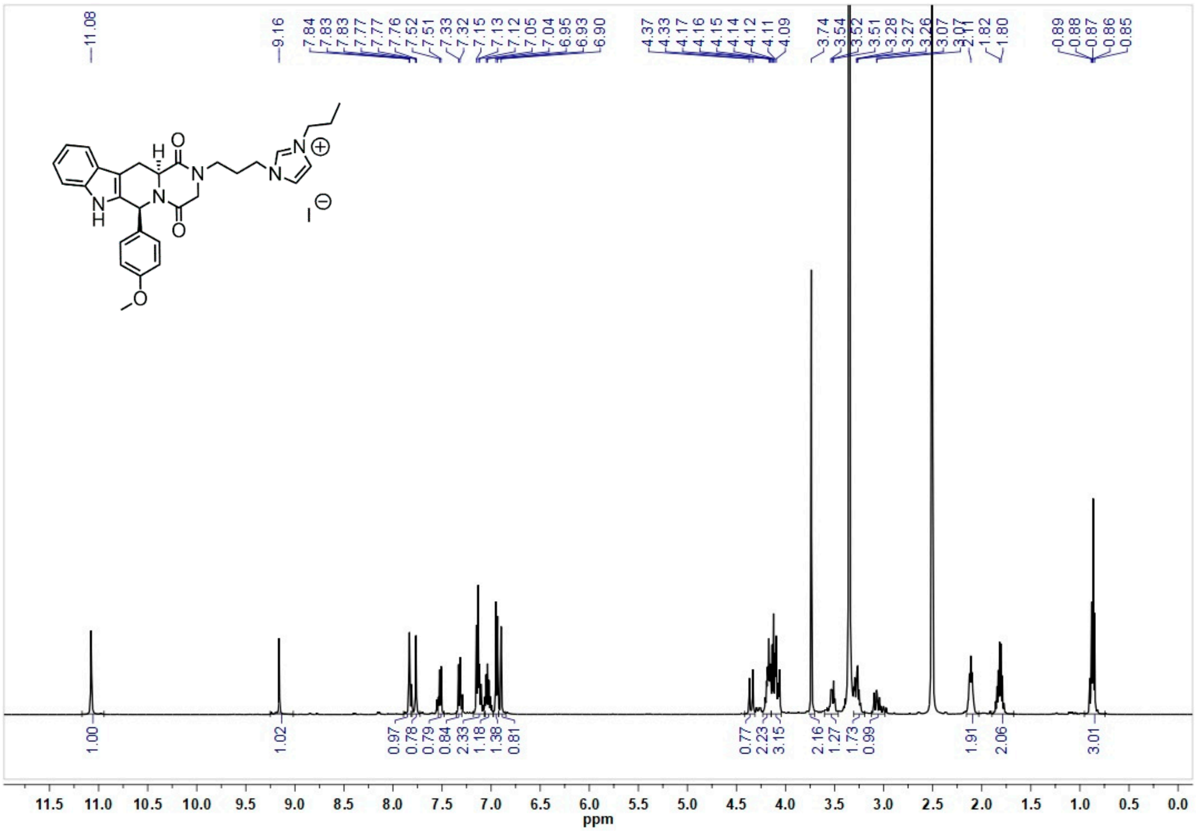

^{13}C NMR spectrum of **12c**

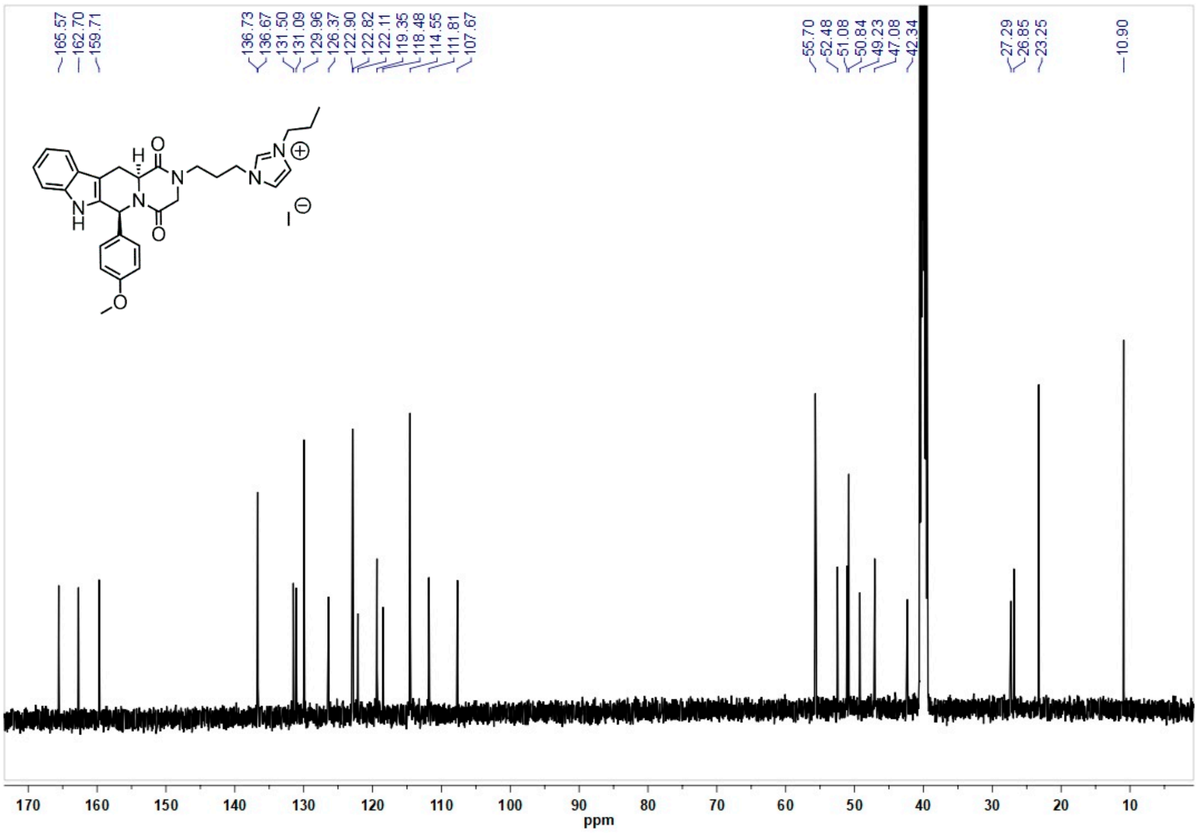

12. NMR spectra of imidazolium salt **12d**

^{1}H NMR spectrum of **12d**

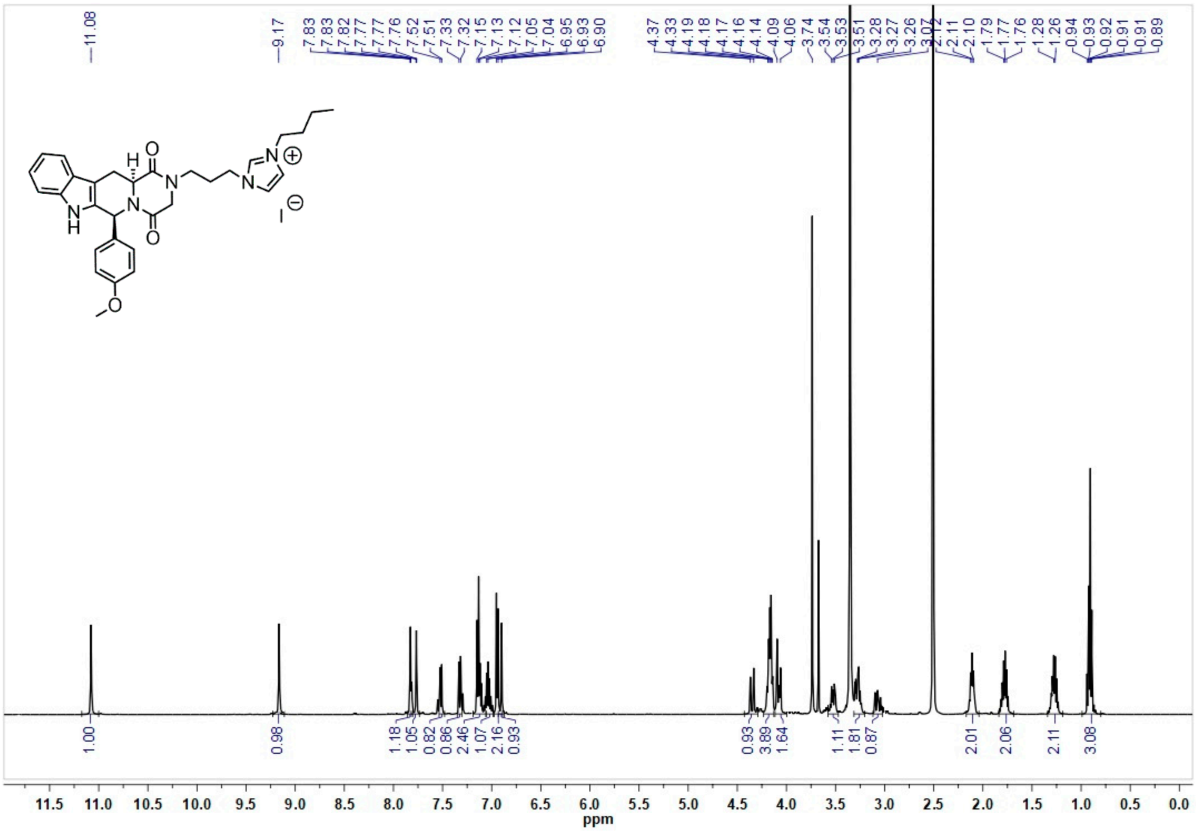

^{13}C NMR spectrum of **12d**

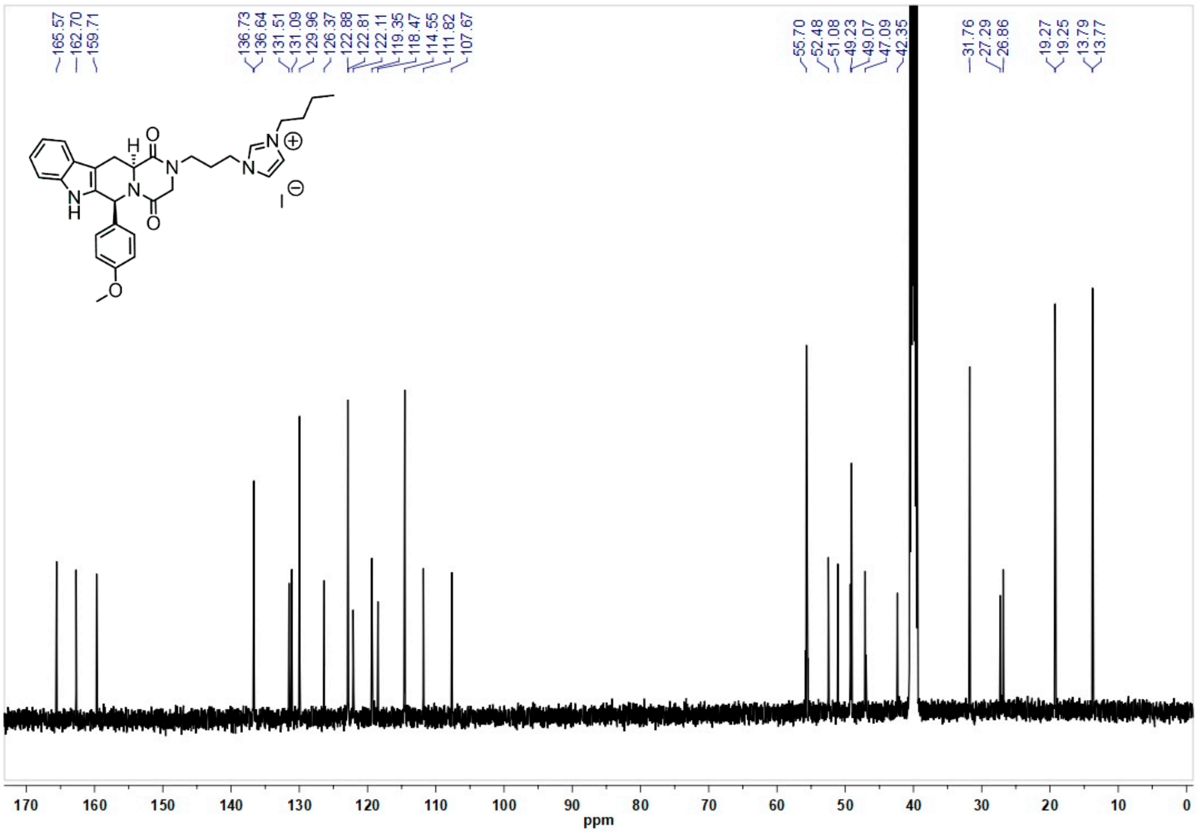

Supplement: Supplementary file 1 [file molecules-31-01563-s001.zip › molecules-4297911-supplementary.pdf]
